# Motion cues from the background influence associative color learning of honey bees in a virtual-reality scenario

**DOI:** 10.1038/s41598-021-00630-x

**Published:** 2021-10-26

**Authors:** Gregory Lafon, Scarlett R. Howard, Benjamin H. Paffhausen, Aurore Avarguès-Weber, Martin Giurfa

**Affiliations:** 1 Research Centre on Animal Cognition, Center for Integrative Biology, CNRS, University Paul Sabatier - Toulouse III, 118 Route de Narbonne, 31062 Toulouse Cedex 09, France; 2 College of Animal Sciences (College of Bee Science), Fujian Agriculture and Forestry University, Fuzhou, 350002 China; 3 Institut Universitaire de France (IUF), Paris, France; 4 Present Address: School of Life and Environmental Sciences, Melbourne Burwood Campus, Deakin University, Melbourne, VIC Australia

**Keywords:** Classical conditioning, Visual system, Zoology

## Abstract

Honey bees exhibit remarkable visual learning capacities, which can be studied using virtual reality (VR) landscapes in laboratory conditions. Existing VR environments for bees are imperfect as they provide either open-loop conditions or 2D displays. Here we achieved a true 3D environment in which walking bees learned to discriminate a rewarded from a punished virtual stimulus based on color differences. We included ventral or frontal background cues, which were also subjected to 3D updating based on the bee movements. We thus studied if and how the presence of such motion cues affected visual discrimination in our VR landscape. Our results showed that the presence of frontal, and to a lesser extent, of ventral background motion cues impaired the bees’ performance. Whenever these cues were suppressed, color discrimination learning became possible. We analyzed the specific contribution of foreground and background cues and discussed the role of attentional interference and differences in stimulus salience in the VR environment to account for these results. Overall, we show how background and target cues may interact at the perceptual level and influence associative learning in bees. In addition, we identify issues that may affect decision-making in VR landscapes, which require specific control by experimenters.

## Introduction

Understanding the spatiotemporal processes that guide decision-making in animals and humans is essential in cognitive research and may be facilitated by virtual reality (VR)^1,2^, which allows generating of immersive spatial environments in well-controlled laboratory settings. In such environments, experiences are simulated based on changes of perceived landscapes or images, which are updated based on the subject’s own movements and decisions^1,2^.

Insects have pioneered the implementation of VR paradigms aimed at studying perceptual and cognitive capacities. A predecessor of current VR systems is the flight simulator conceived for the fruit fly *Drosophila melanogaster*. In this setup, which was first used to study how optical properties of compound eyes influence optomotor reactions^3^, a tethered fly flies stationary in the middle of a cylindrical arena and experiences surrounding visual stimuli that can be updated by the fly’s movements. Newer versions of this apparatus are still used for numerous studies on visual learning and memory in flies^4–7^. ‘Locomotion compensators’ were also developed to study decision-making by walking insects on two-dimensional surfaces. In silk moths and honey bees, for instance, a ‘servosphere’—a form of spherical treadmill that compensates every locomotive movement of a walking insect—was first used to study olfactory orientation towards controlled odor stimuli such as pheromone components and odor gradients^8,9^. Spherical treadmills have been used to study multiple behaviors in different insect species. In these setups, the walking movements of the insect under study are constantly monitored and translated into displacements of surrounding visual cues (closed-loop conditions). The insect can be either free^10,11^ or immobilized^12–16^ by a tether glued onto its body surface (typically on the thorax). In both cases, the insect walks stationary on a treadmill whose movements are recorded by captors placed lateral or ventral to the treadmill.

VR setups are particularly useful for the presentation of visual cues and the study of visual performances. Screens consisting of LED bulb arrays are commonly employed to provide simple forms of visual stimulation^17–19^. In addition, stimuli projected onto screens by high-rate video-projectors have also been used on walking arthropods (e.g.^12–14,16,20^). Furthermore, treadmills holding a tethered animal can also be set in natural visual surroundings to study the influence of landscape features on navigation performances^10^.

Owing to their status of classic models for the study of visual cognition^21–23^, the visual performances of honeybees (*Apis mellifera*) have recently started to be studied in VR setups. The main drive to develop these studies was the impossibility to access the neural underpinnings of visual performances in free-flying bees, which have been traditionally used to study basic properties of visual learning and perception^24^. Immobilized bees have been traditionally required for population recordings of neural activity in the bee brain^25,26^, thus precluding the possibility of recording active visually-driven behaviors. VR setups in which a tethered animal makes decision based on visual cues represent a suitable solution to overcome these limitations as they provide access both to behavioral output and to the nervous system of a behaving bee with restricted mobility^27,28^. This perspective is supported by recent developments allowing to record from specific neurons in the brain of walking bees^16,27,29–31^. Yet, the development of VR environments requires considerable work in order to adapt visual displays to the subjective perception of an insect and determine optimal parameters for immersive sensations from an insect’s perspective.

Prior work allowed the development of virtual-reality (VR) systems in which a tethered honey bee walks stationary on a spherical treadmill (a Styrofoam ball floating on an air cushion) while perceiving a virtual environment displayed by a video projector onto a semicircular screen^12–16,27^. In most cases, however, the visual stimulation provided consisted of a 2D virtual environment in which only translational image movement (left–right) was coupled to the bees’ movements, thus providing an imperfect immersive environment. Despite the absence of depth components, bees learned both elemental (e.g. discrimination between blue vs. green discs or squares)^12–14^ and non-elemental discriminations (e.g. the negative patterning problem in which responding to a visual compound, but not to its components, has to be suppressed)^15^, thus showing the suitability of VR for the study of visual learning.

Here we introduce an improved version of our prior VR setup in which a custom-made software allowed us to create a 3D virtual landscape in which bees move and learn to discriminate visual stimuli. This modification introduced depth perception estimated via the optic flow generated by the bee’s own movements as a new variable, whose influence on the visual discrimination needs to be considered. In this new scenario, motion cues were not only derived from the targets themselves, but also from the background presented either ‘behind’ the vertically displayed targets or ventrally, on the walking surface. We therefore studied if and how the addition of these motion cues to our VR setup affects learning and discrimination in tethered bees.

## Materials and methods

### Study species and collection

Honey bee foragers (*Apis mellifera*) were obtained from the CRCA apiary located in the campus of the University Paul Sabatier. Foragers were captured at gravity feeders providing 0.88 M sucrose solution upon landing and before they began feeding. This step is important as it ensures that only bees with the appropriate appetitive motivation were brought to the laboratory for the visual learning experiments. Captured bees were enclosed in individual glass vials and then transferred to small cages housing ten bees in average; where they had access to ad libitum water and 300 µl of 1.5 M sucrose solution. They were then kept overnight in an incubator at 28 °C and 80% humidity. On the next day, each bee was cooled on ice for 5 min to anesthetize it and attach it to its tether. Bees were handled under red light, which ensured a dark environment to the insects.

### Tethering procedure

**Figure 1:**
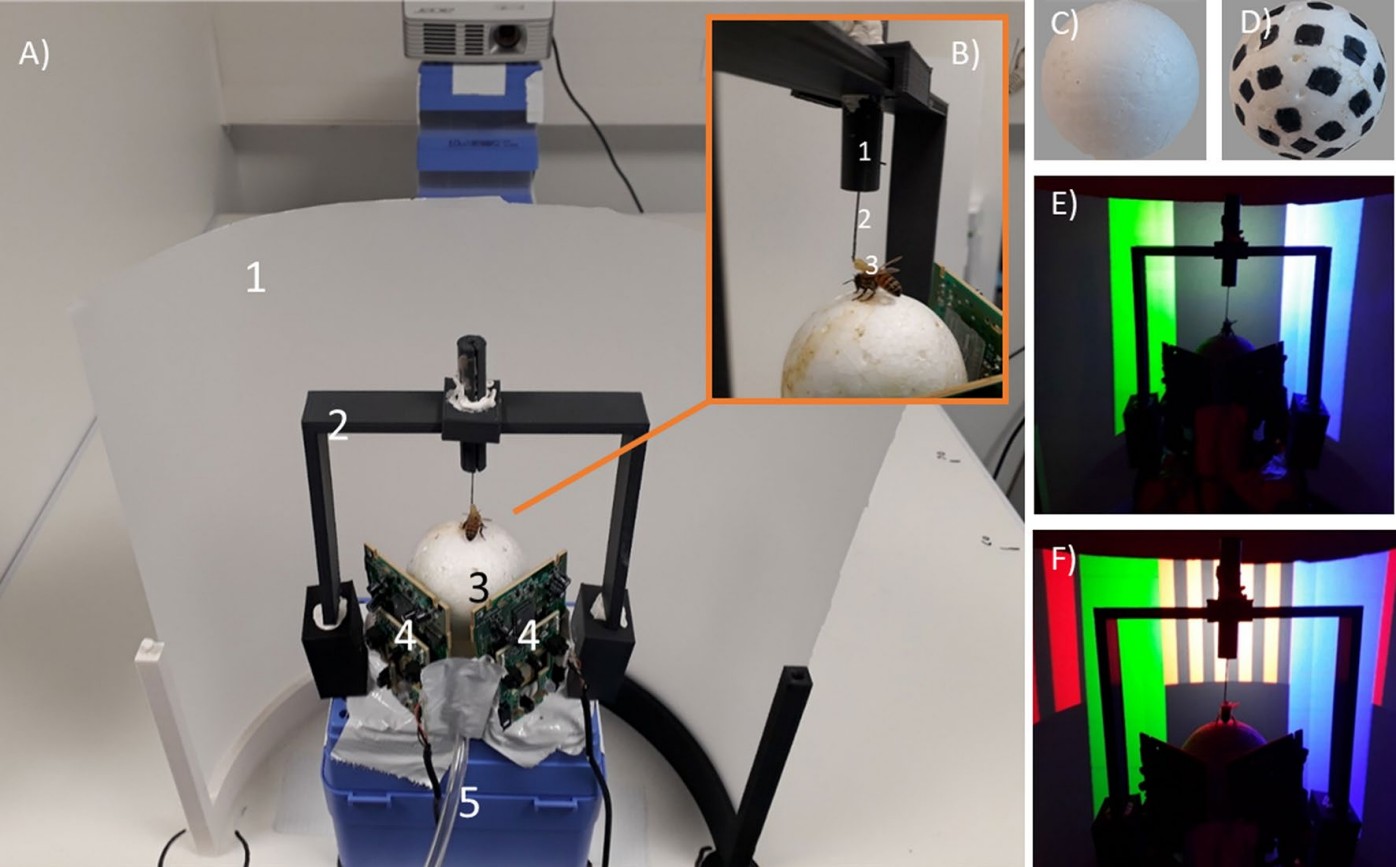
Experimental setup for 3D virtual-reality (VR) studies in honey bees. (**A**) Global view of the VR system. 1: Semicircular projection screen made of tracing paper. 2: Holding frame to place the tethered bee on the treadmill. 3: The treadmill was a Styrofoam ball positioned within a cylindrical support (not visible) and floating on an air cushion. 4: Infrared mouse optic sensors allowing to record the displacement of the ball and to reconstruct the bee’s trajectory. 5: Air arrival. (**B**) The tethering system. 1: Plastic cylinder held by the holding frame; the cylinder contained a glass cannula into which a steel needle was inserted. 2: The needle was attached to the thorax of the bee. 3: Its curved end was fixed to the thorax by means of melted bee wax. (**C**, **D**) Two types of Styrofoam balls used for assessing the importance of the ventral optic flow. (**C**) No ventral optic flow provided. (**D**) Ventral optic flow provided. (**E**) Color discrimination learning in the VR setup. The bee had to learn to discriminate a rewarded from a non-rewarded color cuboid. Cuboids were green and blue. In this case color training and testing was set in the ‘*Transparent Condition*’, i.e. no background was provided and the VR display contained only the two colored cuboids on an empty dark background. (**F**) Same as in (**E**) but in this case, the vertical background of the VR arena was covered by a vertical grating made of black and reddish bars. Depending on its movements, the background gave origin to three different conditions**:** the ‘*Vertical Grating—Optic Flow Condition*’, in which the grating was set in closed loop conditions with respect to the bee movements; the ‘*Vertical Grating—No Optic Flow Condition*’, in which the grating was moved in synchrony with the bee’s gaze so that no motion cues could be derived from the background; and the ‘*Rotating Vertical Grating Condition*’, in which the grating was displaced in the anti-clockwise direction across the screen at a constant speed, thus generating a constant optic flow that was independent of the bee’s movements.

Each bee was tethered by means of a 0.06 g steel needle, 0.5 mm in diameter and 40 mm in length, which was fixed to the thorax by melted beeswax. The needle was placed within a glass cannula, 1 mm in diameter, which was held within a black plastic cylinder, 1 cm in diameter and 55 mm in length, which was fixed on a holding frame placed above the treadmill (Fig. 1A,B). This system allowed the bee to adjust its position in the vertical axis once set on the ball, but did not allow rotational movements. The holding frame consisted of a vertical black, plastic half frame made of two vertical rectangular supports, 105 mm in length, connected to an upper, horizontal rectangular support, 120 mm in length. The latter held the black cylinder in the middle (Fig. 1B). After being attached to its tether, each bee was placed on a small (49 mm diameter) Styrofoam ball for familiarization to a provisory set-up and provided with 5 μl of 1.5 M sucrose solution. Each bee was held for 3 h in this provisory setup, which was kept in the dark and without visual stimulations.

### Virtual reality set-up

The bee was then moved to the VR setup to be trained and tested in a 3D visual environment. To establish this environment, we used a custom software developed using the Unity engine (version 2018.3.11f1), open-source code available at https://github.com/G-Lafon/BeeVR. The software updated the position of the bee within the VR every 0.017 s.

The VR apparatus consisted of a spherical Styrofoam ball, which acted as a treadmill onto which a stationary bee walked while perceiving an artificial visual landscape displayed in front of it on a semi-circular screen (Fig. 1A). The ball was 50 mm in diameter and weighted 1.07 g (Fig. 1B). The ideal weight (M) for spheres holding insects walking on locomotion compensators was suggested to be^10^ M_sphere_ = 2.5*M_animal_, which in case of a honeybee weighting in average 0.09 g yields a sphere weight of 0.23 g. Despite the fact that our sphere was about 5 times heavier, the bees used in our experiments walked on it without noticeable problems. The ball was positioned within a 3D-printed, hollow, cylindrical support (cylinder: 50 mm high, 59 mm diameter). The cylinder allowed distributing an upwards air flow of 33 L min^−1^ produced by an AquaOxy 2000 aquarium pump, and released through a small hole at the base of the cylindrical support. The Styrofoam ball floated on the resulting air cushion and the tethered bee walked on it while remaining stationary. If the bee moves forward, the ball moved backwards and if it intended to turn to the right or the left, the ball moved to the left or the right, respectively. The ball was white and unmarked (Fig. 1B,C) except in the experiment where the influence of the ventral optic flow was tested. In this case, we compared the bees’ performance using a white ball (Fig. 1C) and a ball displaying a black and white checkered pattern made of 7 mm^2^ squares (Fig. 1D). The movements of the ball, and thus the walking behavior of the bee (i.e. speed, orientation and location in the virtual environment), were recorded by two infrared optic-mouse sensors (Logitech M500, 1000 dpi) placed at a distance of 7 mm from the sphere and forming an angle of 90° angle relative to each other (i.e. 45° from the bee body axis; see Fig. 1A).

The ball was positioned in front of a half-cylindrical vertical screen, 268 mm in diameter and 200 mm height, which was placed at 9 cm from the bee. The screen was made of semi-transparent tracing paper, which allowed presentation of a 180° visual environment to the bee (Fig. 1A). The visual environment was projected from behind the screen using a video projector connected to a laptop (Fig. 1A). The video projector was an Acer K135 (Lamp: LED, Maximum Vertical Sync: 120 Hz, Definition: 1280 × 800, Minimum Vertical Sync: 50 Hz, Brightness: 600 lumens, Maximum Horizontal Sync: 100 × 10^3^ Hz, Contrast ratio: 10,000:1, Minimum Horizontal Sync: 30 × 10^3^ Hz). The lag between the motion of the bee and the update of the visual surrounding was measured by a high-speed camera at 1000 fps (Canon RX10 mkIII). The VR display started as usual and the hovering motionless ball was quickly moved by hand. A high-speed video containing the ball, the hand and the VR was shot. The number of frames until the background illumination changed were counted by two researchers independently. This procedure yielded a lag value of 18.00 ± 2.53 ms (mean ± S.E.; n = 10).

### Experiment 1: choosing the red intensity for the achromatic black–red background gratings

Honey bees can perceive a red target in achromatic terms and can discriminate it from black based on its achromatic L-receptor contrast^32,33^. In order to present a vertical, red and black striped background against which a color discrimination had to be achieved, we first performed an experiment to choose the intensity of red that was most appropriate for our background grating. We thus determined the spontaneous phototactic responses of bees towards a vertical red cuboid, which varied in intensity. A red intensity that was high enough to be perceived should induce phototactic attraction.

**Figure 2:**
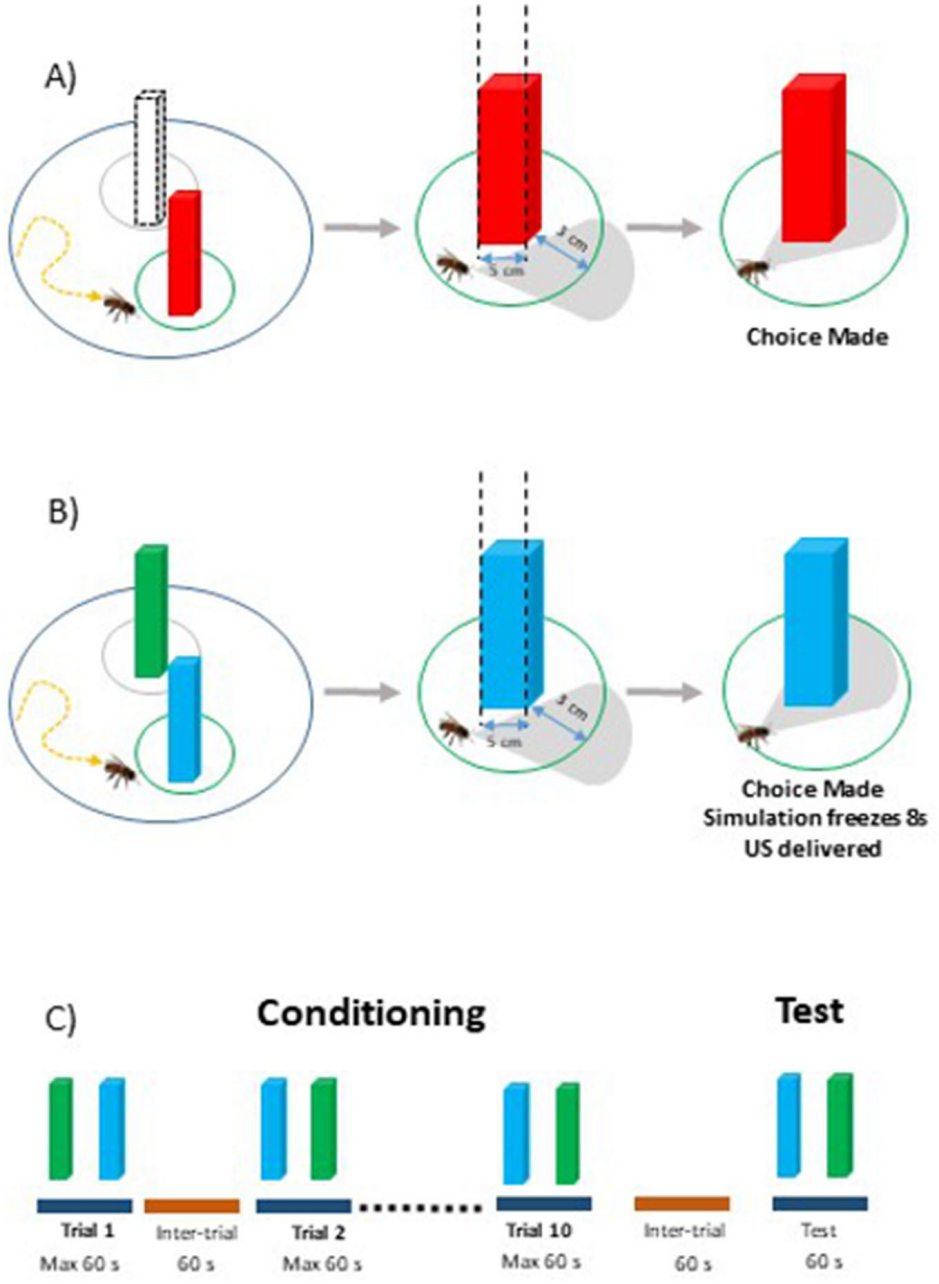
Choice and discrimination learning tasks in the VR setup. (**A**) Experiment 1: Quantification of the spontaneous phototactic responses of bees towards a red cuboid against the absence of an equivalent stimulus in the symmetric position (dashed cuboid). Choice of the red cuboid was recorded if the bee reached a virtual area of a radius of 3 cm centered on the cuboid and fixed it frontally. (**B**) Experiments 2 and 3: Color discrimination learning with a green and a blue cuboid. One cuboid was rewarded with sucrose solution and the other punished with either quinine solution (Experiment 2) or saline solution (Experiment 3) delivered by the experimenter. A choice was recorded when the bee reached an area of a radius of 3 cm centered on the cuboid and fixed it frontally. The cuboid image was then frozen during 8 s and the corresponding reinforcement (US) was delivered. (**C**) Experimental schedule of color learning experiments (Experiments 2 and 3). Bees were trained along 10 conditioning trials that lasted a maximum of 1 min and that were spaced by 1 min (intertrial interval). After the end of conditioning, and following an additional interval of 1 min, bees were tested in extinction conditions with the two colored cuboids during 1 min.

The cuboid had a 5 × 5 cm base and 1 m height so that it occupied the entire vertical extent of the screen irrespective of the bee’s position (Fig. 2A, left). At the beginning of each trial, it subtended a horizontal visual angle of 6.5° and was positioned either to the left (− 50°) or the right (+ 50°) of the tethered bee. Approaching the cuboid resulted in an expansion of its horizontal extent (1.7°/cm). A choice was recorded when the bee approached the cuboid within an area of 3 cm surrounding its virtual surface and directly faced its center (Fig. 2A, middle and right). Three different groups of bees were tested, each one with a different red intensity (see Fig. S1A): Red 10 (RGB: 26, 0, 0; irradiance: 13 μW/cm^2^; N = 19), Red 50 (RGB: 128, 0, 0; irradiance: 140 μW/cm^2^; N = 19) or Red 100 (RGB: 255, 0, 0; irradiance: 1130 μW/cm^2^; N = 20). Table 1 summarizes the conditions of this Experiment as well as those of the subsequent experiments.

**Table 1:** Summary of the experimental conditions provided in Experiments 1 to 3.

Experiment	Condition training—test	Background	*N*
Experiment 1 Choice of red intensity	*Red 10* No training—test: red 10 versus nothing	Frontal black	19
	*Red 50* No training—test: red 50 versus nothing	Frontal black	19
	*Red 100* No training—test: red 100 versus nothing	Frontal black	20
Experiment 2 Frontal motion cues	*Transparent Condition* Training & test: blue versus green	Frontal black	24
	*Vertical Grating—Optic Flow Condition* Training & test: blue versus green	Frontal: black & red vertical stripes closed loop	17
	*Vertical Grating—No Optic Flow Condition* Training & test: blue versus green	Frontal: black & red vertical stripes fixed to the Bee’s gaze	17
	*Rotating Vertical Grating Condition* Training & test: blue versus green	Frontal: black & red vertical stripes constantly rotating	17
Experiment 3 Ventral motion cues	*No Ventral Optic Flow Condition* Training & test: blue versus green	Frontal: black Ventral: none (white treadmill)	29
	*Ventral Optic Flow Condition* Training & test: blue versus green	Frontal black Ventral: (black and white treadmill)	38

N: sample size of each condition.

Within each group, each bee was subjected to four consecutive tests in extinction conditions. During a test, the bee faced the red cuboid on one side, and no stimulus on the alternative side. We recorded whether the bee chose the red cuboid or the equivalent empty space on the other side (to account for possible stimulus choice from random locomotion paths). Each test lasted 60 s and the inter-test interval was 10 s.

### Experiment 2: the influence of motion cues from a vertical background on color discrimination

Having chosen a red intensity for the red and black striped vertical background (Red 100; see above), we trained bees to discriminate between two vertical colored cuboids, one rewarded and the other not (see below). Both cuboids had the same dimensions of the red cuboid employed in the previous experiment. One was blue (RGB: 0, 0, 255, with a dominant wavelength of 446 nm) and the other green (RGB: 0, 51, 0, with a dominant wavelength at 528 nm) (Figs. 1E, 2B left) (see Fig. S1A). Their intensity, measured at the level of the bee eye, was 161,000 μW/cm^2^ (blue cuboid) and 24 370 μW/cm^2^ (green cuboid). These values were shown to elicit the same level of spontaneous attraction^12,15^. The cuboids were positioned respectively at − 50° and + 50° from the bee’s body axis at the beginning of each trial. As in the previous experiment, approaching a cuboid within an area of 3 cm surrounding its virtual surface followed by direct fixation of its center was recorded as a choice (Fig. 2B middle and right).

The background on which the color cuboids were visible was varied to assess the effect of background motion cues on visual discrimination learning. Four experimental conditions were defined (see Table 1). In the ‘*Transparent Condition*’ (N = 24), no background was provided and the VR display contained only the two cuboids on an empty dark background (Fig. 1E). The residual light from the empty background had a dominant wavelength of 449 nm and an irradiance of 38 µW/cm^2^. In the ‘*Vertical Grating—Optic Flow Condition*’ (N = 17), the walls of the virtual arena were covered by a vertical grating made of black (RGB: 0, 0, 0; irradiance: 45 μW/cm^2^; dominant wavelength 628 nm) and red bars (RGB: 255, 0, 0; irradiance: 1130 μW/cm^2^; dominant wavelength 628 nm), each subtending a visual angle of 6° (Fig. 1F). Moving forward increased this visual angle by 0.18°/cm. In the ‘*Vertical Grating—No Optic Flow Condition*’ (N = 17), the same grating made of black and red bars was used but the VR software moved it in synchrony with the bee’s gaze so that no motion cues could be derived from the background. Finally, in the ‘*Rotating Vertical Grating Condition*’ (N = 17), the same black and red grating was displaced in the anti-clockwise direction across the wall at a constant speed (12 m/s), thus generating a constant optic flow that was independent of the bee’s movements.

### Experiment 3: the influence of motion cues from a ventral background on color discrimination

In order to test the potential impact of ventral motion cues, we trained bees to discriminate the same two vertical colored cuboids used in the previous experiment under two different conditions. While the vertical frontal background remained the same as in the *Transparent Condition* of Experiment 2 (Fig. 1E), the treadmill texture was varied between two groups of bees: in one case it was a plain white surface (Fig. 1C; N = 29) while in the other case, it was a black and white checkered pattern made of 7 mm^2^ squares (Fig. 1D; N = 38). While the first condition did not provide ventral optic flow, the second condition provided it (see Table 1).

### Training and testing procedure for the Experiments 2 and 3

Bees were trained during 10 trials using a differential conditioning procedure (Fig. 2C) in which one of the cuboids (i.e. one of the two colors, green or blue) was rewarded with 1.5 M sucrose solution (the appetitive conditioned stimulus or CS+) while the other cuboid displaying the alternative color (the aversive conditioned stimulus or CS−) was associated with either 60 mM quinine (Experiment 2)^34^ or 3 M NaCl solution^35,36^ (Experiment 3). The latter was used to increase the penalty for incorrect choices^37^.

At the beginning of the experiment, bees were presented with a dark screen for 60 s. During training trials, each bee faced the virtual environment with the two cuboids in front of it. The bee had to learn to choose the CS+ cuboid by walking towards it and centering it on the screen. Training was balanced in terms of color contingencies (i.e. blue and green equally rewarded across bees) based on a random assignment by the VR software. If the bee reached the CS+ within an area of 3 cm in the virtual environment (i.e. the chosen cuboid subtended a horizontal visual angle of 53°) and centered it in its front, the screen was locked on that image for 8 s (Fig. 2B). This allowed the delivery of sucrose solution in case of a correct choice, or of quinine or NaCl in case of an incorrect choice. Solutions were delivered for 3 s by the experimenter who sat behind the bee and used a toothpick to this end. The toothpick contacted first the antennae and then the mouthparts while the screen was locked on the visual image fixated by the bee.

Each training trial lasted until the bee chose one of both stimuli or until a maximum of 60 s (no choice). Thus, a single choice (or a no choice) was recorded during each training trial. Trials were separated by an inter-trial interval of 60 s during which the dark screen was presented. The bees that were unable to choose a stimulus in at least 5 trials were excluded from the analysis. From 216 bees trained in the Second Experiment, 75 were kept for analysis (~ 35%). From 272 bees trained in the Third Experiment, 67 bees were kept for analysis (~ 25%).

After the last training trial, each bee was subjected to a non-reinforced test (Fig. 2C) that contrary to training trials had a fixed duration of 60 s. During this test, two variables were recorded: the first choice (as defined above) and the time spent fixating the rewarded and the non-rewarded stimulus. Both variables have been used in prior works performed in our VR setup to characterize test performances as they may reveal different aspects of behavioral performances^12,13,15^. Fixation time (s) was defined as the time spent by each cuboid at the center of the screen (± 2.5 mm) where it was brought by the bee’s motor actions. We used a ray-casting approach to determine if the object was there and recorded collisions between a ray following the forward vector of the bee and the center of the object.

### Statistical analysis

Statistical analyses were performed using R software^38^. In Experiment 1 (red perception), the first choice of the bees in each test was categorized according to three mutually exclusive categories: Red Stimulus (Red), No Stimulus (NS: choice of the area symmetric to the stimulus position) and no choice (NC). Individual choices were translated into a binomial format (0 or 1) within each category. For instance, a bee choosing the red cuboid was recorded as (1, 0, 0) for a choice of the red stimulus, choice of the no stimulus and NC, respectively. In Experiments 2 and 3, the first choice in each trial and test was categorized as choice of the CS+, choice of the CS− or no choice (NC). Thus, a bee choosing the CS+ was recorded as (1, 0, 0) for choice of the CS+, choice of the CS− and NC, respectively. Data were bootstrapped to plot the proportion of bees in each category with their corresponding 95% confidence interval. Performances were analysed using generalized mixed linear models (GLMM) with a binomial error structure-logit-link function (glmer function of R package lme4)^39^. The independent variables (fixed factors) were the experimental group (*Condition*), the trial number (*Trial;* Experiments 2 and 3), the choice category (*Choice*) and the color of the CS+ when applicable (*Color*: Blue or Green). *Bee ID* was included as a random factor to account for the repeated-measure design. Several models were run by testing interactions between factors and by dropping each factor subsequently to select the model with the highest explanatory power (i.e. the lowest AIC value). *P* values for each factor or interaction were obtained by comparing models. The Tukey method was used for multiple comparisons within the selected model; z values are reported for these analyses. For all experiments, the modeling results are reported in Tables S1 to S3 in Supplementary Information. During the tests of Experiments 2 and 3, we also recorded the time spent fixating the test alternatives (CS+ vs. CS−). Time values were compared using a Wilcoxon signed rank test.

For the acquisition trials, we recorded motor variables such as the total distance walked during a trial, the walking speed, and the tortuosity of the trajectories. Tortuosity was calculated as the ratio between the total distance walked and the distance between the first and the last point of the trajectory connected by an imaginary straight line. When the ratio was 1, or close to 1, trajectories were straightforward while higher values corresponded to sinuous trajectories. In addition, we analyzed the latency to make a choice starting from the beginning of a trial to the moment in which a choice (either for the CS+ or the CS−) was recorded. NC data were excluded from the latency analysis. The analysis of these continuous variables was done using a linear mixed model (lmer function) in which the individual identity (*Bee ID*) was a random factor and the experimental condition (*Condition*) and trial number (*Trial*) were fixed factors.

For each experimental condition, we represented the bees’ cumulative trajectories (CS+ choosing and CS− choosing bees) in terms of heat maps, which show the cumulative coordinates occupied by the bees either during the ten training trials or during the non-reinforced test to which they were subjected. Coordinates were binned into 1 cm^2^. Warmer colors depict locations more frequently occupied (see color bar). The highest frequency is cut down to 10% of the maximum on the color bar. This was done to decrease the excessive occupancy frequency of the starting point at the expense of other locations, given that it was the same for all bees. While the side of the rewarded stimulus was randomized, it was placed arbitrarily on the left in the heat maps.

## Results

### Experiment 1: choosing the red intensity for the achromatic black and red background

**Figure 3:**
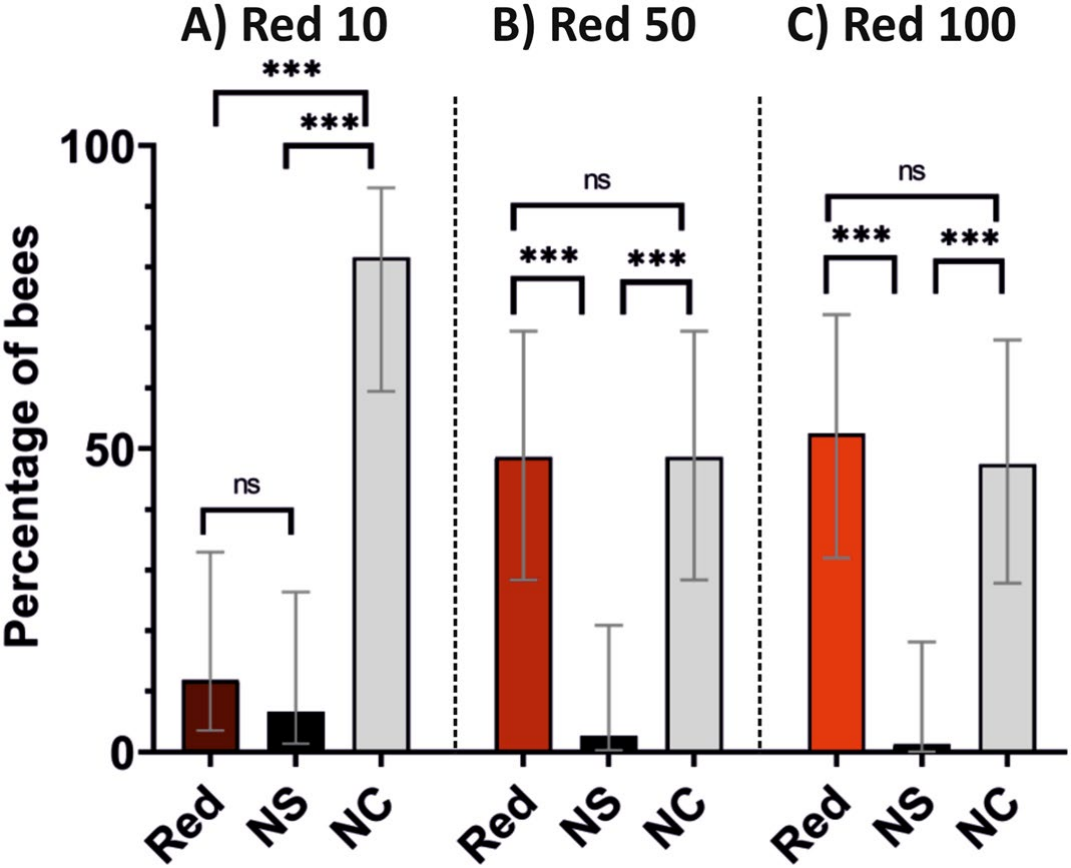
Experiment 1—Choosing the red intensity for the achromatic black and red background. Quantification of the spontaneous phototactic responses of bees towards a red cuboid (see Fig. 2A). Three different intensities were assayed, each with a different group of bees: Red 10 (RGB: 26, 0, 0; irradiance: 13 μW/cm^2^; N = 19), Red 50 (RGB: 128, 0, 0; irradiance: 140 μW/cm^2^; N = 19) and Red 100 (RGB: 255, 0, 0; irradiance: 1130 μW/cm^2^; N = 20). For each intensity, the figure represents the pooled performance of four consecutive extinction tests in which the spontaneous attraction towards the red cuboid (‘Red’) was quantified. ‘NS’ (no stimulus) represents the choice of an equivalent empty area in the VR arena that was opposite to the red cuboid (see Fig. 2A). NC: no choice. Both the Red-50 and the Red-100 intensities were sufficient to render the red stimulus detectable for honeybees. For subsequent experiments, the Red 100 intensity was chosen.

In a first experiment, we determined the spontaneous phototactic responses of bees towards a red cuboid (dominant wavelength 628 nm) varying in intensity. Using different groups of bees, we tested three different intensities to define the one that would be sufficient to induce phototactic attraction: Red 10 (RGB: 26, 0, 0; irradiance: 13 μW/cm^2^), Red 50 (RGB: 128, 0, 0; irradiance: 140 μW/cm^2^) and Red 100 (RGB: 255, 0, 0; irradiance: 1130 μW/cm^2^). Each bee was tested along four consecutive tests with the same intensity. The model that best fitted the data included an interaction between the red intensity and the bees’ choice (Choice*Intensity: *χ*^2^ = 65.48, df: 4, *p* < 0.001). There was no significant effect of the test sequence on the bees’ choices (*Test*: χ^2^ = 0.002, df:1, *p* = 0.97). Thus, we pooled the data of the four tests and represented for each intensity the percentage of bees within each category (Red, No Stimulus and No Choice; Fig. 3).

In the Red 10 condition (Fig. 3A; N = 19 bees), the bees did not prefer the red cuboid to the alternative symmetrical area displaying no stimulus (Red 10 vs. NS: 11.8% vs. 6.8% of choices, z = 1.11, *p* = 0.27). Most of the bees did not choose in this condition (81.6% of the cases: NC vs. Red 10: z = 7.56, *p* < 0.0001; NC vs. NS: z = 7.54, *p* = < 0.0001). By contrast, both in the Red 50 (Fig. 3B; N = 19) and in the Red 100 condition (Fig. 3C; N = 20), bees preferred the red stimulus to the equivalent area displaying no stimulus (Red 50 vs. NS: 48.7% vs. 2.6%, z = 4.73, *p* < 0.0001; Red 100 vs. NS: 52.5% vs. 1.3%, z = 4.34, *p* < 0.0001). The percentage of bees not choosing remained high and similar to that of bees choosing the red cuboid (Red 50 vs. NC: z = 0.000, *p* = 1.00; Red 100 vs. NC: z = 0.58; *p* = 0.53). For both intensities, the proportion of non-choosing bees was significantly higher than the choice of the absence of stimulus (Red 50 vs. NS: 48.7% vs. 48.6%, z = 4.73 *p* < 0.0001; Red 100 vs. NS: 52.5% vs. 47.5%, z = 4.14 *p* < 0.0001). These results indicate that both the Red-50 and the Red-100 intensities were sufficient to render the red stimulus detectable for honeybees. We therefore chose the Red-100 intensity for the red-and-black gratings used in the subsequent experiment as it was the more salient stimulus from the two that were detectable by the bees. We were confident that Red 100 would not induce higher phototaxis than Red 50 as no differences in attraction existed between the cuboids displaying these two lights (compare Fig. 3B,C).

### Experiment 2: the influence of motion cues from a vertical frontal background on color discrimination

Four different frontal background conditions were used to assess the effect of motion cues from the background during color discrimination learning. In the ‘*Transparent Condition*’ (N = 24 bees), the blue and green cuboids were displayed against a uniform dark background. In the ‘*Vertical Grating—Optic Flow Condition*’ (N = 17 bees), the cuboids were presented against a red-and-black vertical grating, which was coupled to the bee’s movements (closed-loop conditions). In the ‘*Vertical Grating—No Optic Flow Condition*’ (N = 17 bees), the cuboids were displayed against the same red-and-black grating but motion cues from the background were suppressed by keeping it constantly fixed to the bee’s gaze. Finally, in the ‘*Rotating Vertical Grating Condition*’ (N = 17 bees), the cuboids were shown against the same red-and-black grating, which was rotated counterclockwise around the virtual arena at a constant speed, thus generating a constant optic flow even when the bee did not move.

### Discrimination learning during training

**Figure 4:**
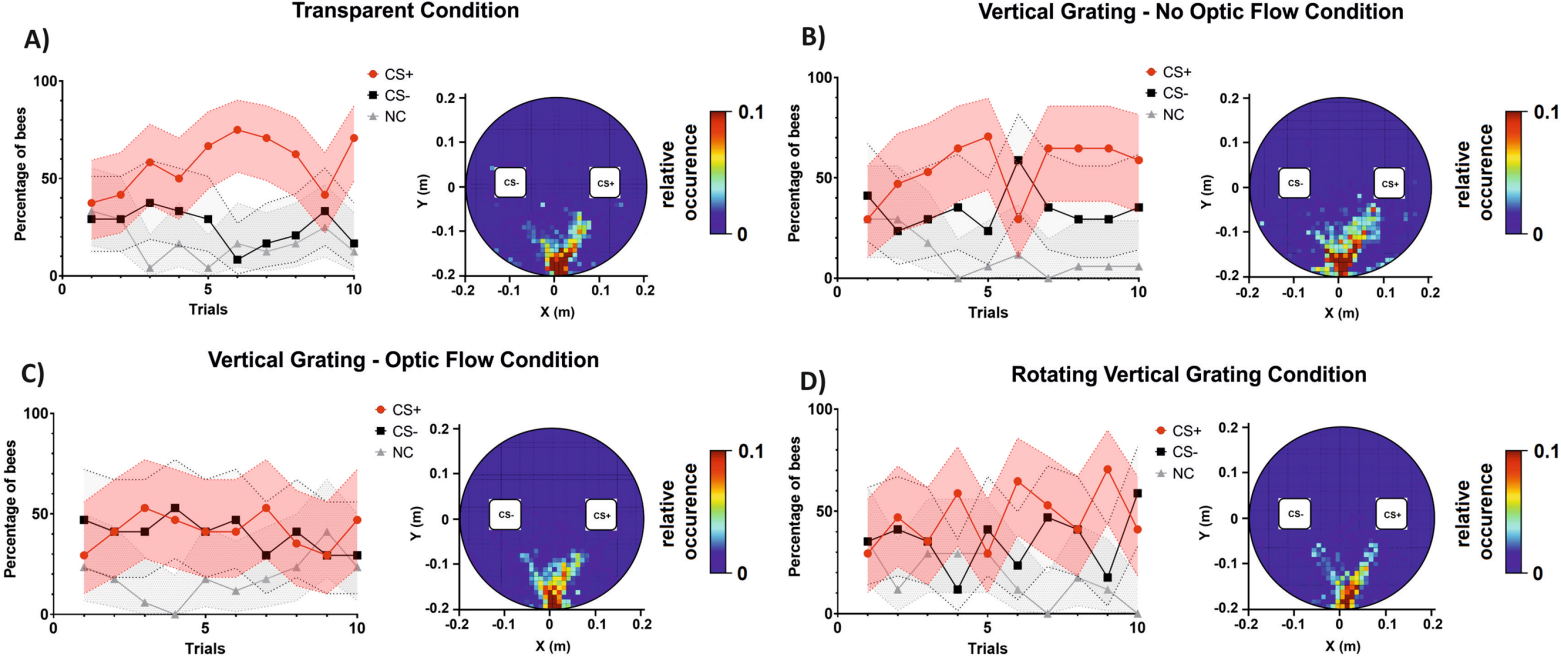
Acquisition performances in a color discrimination learning task under four different background conditions. Each panel shows on the left the acquisition curves in terms of the percentage of bees responding to the CS+ (red), to the CS− (black) or not making any choice (NC; gray) during the ten conditioning trials. The pink, light gray and gray areas around the curves represent the 95% confidence interval of CS+, CS− choices and NC, respectively. On the right of each panel, a heat map shows the cumulative coordinates occupied by the bees trained under each background condition during the ten training trials. Coordinates were binned into 1 cm^2^. Warmer colors depict locations more frequently occupied (see color bar). The highest frequency is cut down to 10% of the maximum on the color bar. While the side of the rewarded stimulus was randomized along conditioning trials, it was placed arbitrarily on the right in the heat maps. (**A**) In the ‘*Transparent Condition*’ (N = 24), the blue and green cuboids were displayed against a dark background. (**B**) In the ‘*Vertical Grating—No Optic Flow Condition*’ (N = 17), the cuboids were displayed against the same red-and-black grating but motion cues from the background were suppressed by keeping it constantly fixed to the bee’s gaze. (**C**) In the ‘*Vertical Grating –Optic Flow Condition*’ (N = 17), the cuboids were presented against a red-and-black vertical grating, which was coupled to the bee’s movements (closed-loop conditions). (**D**) In the ‘*Rotating Vertical Grating Condition*’ (N = 17), the cuboids were shown against the same red-and-black grating, which was rotated counterclockwise around the virtual arena at a constant speed, thus generating a constant optic flow even when the bee did not move.

Figure 4A–D shows the learning curves of the four groups of bees trained to discriminate the green from the blue cuboid under different background conditions and the cumulative heat maps displaying the locations of the bees in their trajectories during the ten acquisition trials. Learning curves were obtained by recording the percentage of bees choosing correctly the CS+ or the CS− in their first choice, or not choosing any stimulus (NC) during each trial. The best explanatory model of the acquisition performance included a three-way interaction between the condition, the trial number and the bees’ choice (χ^2^ = 50.11, df:15, *p* < 0.001) but no effect of the nature of the CS+ was found (blue or green: χ^2^ = 0.000, df:1, *p* = 1). For each background condition, data were thus represented as a CS+ vs. a CS− discrimination irrespective of color identity. In the heat maps, the rewarded cuboid is represented on the left side although its side was randomized along the training sequence.

When no grating was present in the background and the colored cuboids were displayed against a dark homogeneous background (‘*Transparent Condition*’; Fig. 4A), bees learned to respond more to the CS+ than to the CS−. The interaction between trial number and bee choices was significant (χ^2^ = 7.99, df:2, *p* = 0.02). In the course of the 10 conditioning trials, the percentages of bees responding to the CS+ and that of bees responding to the CS− evolved differently (z = 2.51, *p* = 0.01), thus showing successful discrimination learning. Moreover, the dynamic of CS+ responding bees was also significantly different from that of the non-responding (NC) bees (z = 2.17, *p* = 0.03) while the difference between the dynamic of the CS− responding bees and the NC bees was not different (z = 0.13, *p* = 0.9). In the corresponding cumulative heat map, a clear V shape is visible, indicating that the bees did interact equally with both sides in the VR and walked towards the cuboids.

When the red-and-black grating was moved in synchrony with the bee’s gaze so that no motion cues could be derived from the background (‘*Vertical Grating—No Optic Flow Condition*’; Fig. 4B), bees did not modify significantly their stimulus choice along trials. There was a significant interaction between trial number and bee choices (χ^2^ = 13.6, df:2, *p* = 0.001) but only because of a difference in the dynamic of the NC bees compared to other two categories (NC vs. CS+ : z = 3.44, *p* < 0.001; NC vs. CS− : z = 2.60, *p* < 0.001). Although the CS+ and the CS− curves seem to indicate color discrimination, no differences between the dynamics of the percentages CS+ and CS− choosing bees could be detected (z = 1.20, *p* = 0.23), probably because of the high overlap in confidence intervals of these curves. The cumulative heat map representing the locations of the bees during their training trajectories shows that, as in the previous two conditions, bees walked and interacted equally with both sides in the VR.

In the ‘*Vertical Grating—Optic Flow Condition*’ (Fig. 4C), the closed loop conditions included both the cuboids and the background grating, i.e. the bees’ movements translated and expanded not only the cuboids but also the background grating accordingly. The interaction between trial number and bee choices was not significant in this case (χ^2^ = 5.16, df:2, *p* = 0.08). Contrarily to the previous condition, bees were unable to learn the difference between the CS+ and the CS− as no improvement could be detected along the 10 training trials (z = 0.33, *p* = 0.74). Only the dynamics of the non-responding bees was significantly lower than that of bees selecting either the CS+ (z = 4.63, *p* < 0.001) or the CS− (z = 4.33, *p* < 0.001). The cumulative heat map representing the locations of the bees during their training trajectories shows that, as in the previous condition, bees walked towards the cuboids. This result indicates that despite interacting with the cuboids, bees had their color learning impaired by the addition of motion cues from the background.

Finally, in the ‘*Rotating Vertical Grating Condition’* (Fig. 4D) in which the black-and-red grating was displaced at a constant speed irrespective of the bee movements and gaze, a similar pattern than for the *‘No Optic Flow Condition’* was observed. A significant interaction between trial number and bee choices was found (χ^2^ = 11.21, df:2, *p* = 0.004). Yet, it was again due to differences in the dynamic of the percentage of NC bees vs. the percentages of CS+ and CS− bees (NC vs. CS+ : z = 3.11, *p* = 0.002; NC vs. CS− z = 2.71, *p* = 0.007). The percentage of bees choosing the CS+ and that of bees choosing the CS− did not evolve differently (z = 0.44; *p* = 0.66). In this condition, the cumulative heat map shows that bees also walked and interacted equally with the two cuboid sides along trials.

### Motor and temporal components of bee trajectories during training

**Figure 5:**
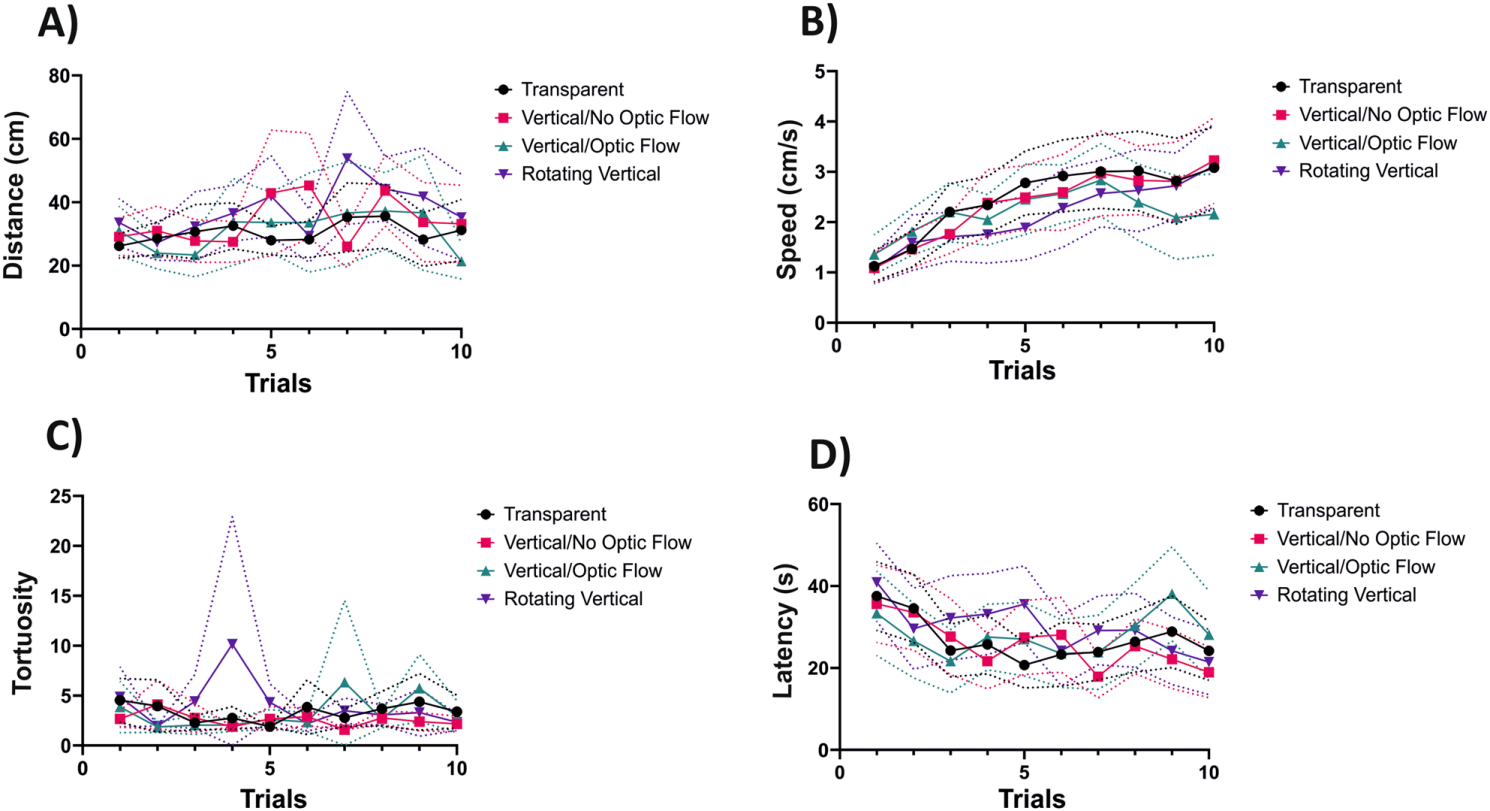
Motor and temporal components of bee trajectories during the acquisition trials. For each background condition, the evolution of (**A**) the distance walked, (**B**) the walking speed, (**C**) the tortuosity and (**D**) the choice latency during training trials is shown. The tortuosity was the ratio between the total distance walked and the straight line connecting the first and the last point of the trajectory during a training trial. *Transparent Condition*’ (N = 24), ‘*Vertical Grating—Optic Flow Condition*’ (N = 17), ‘*Vertical Grating—No Optic Flow Condition*’ (N = 17), ‘*Rotating Vertical Grating Condition*’ (N = 17). The dashed lines above and below the curves represent the 95% confidence interval.

We analyzed if and how motion cues from the background affected the displacement of bees during the training trials in our four background conditions (Fig. 5). To this end, we quantified the distance walked, the walking speed and the tortuosity of the trajectories (ratio between the total distance walked and the straight line connecting the first and the last point of the trajectory). We also measured the choice latency in each trial, i.e. the time required to choose a cuboid within a trial.

The distance walked (Fig. 5A) increased slightly, yet significantly, over trials (*Trial*: χ^2^ = 6.86, df:1, *p* = 0.009) but was not significantly affected by the background condition (*Condition*: χ^2^ = 5.34, df:3, *p* = 0.15). The walking speed (Fig. 5B) also increased during successive trials (*Trial*: χ^2^ = 172.9, df:1, *p* < 0.001) and revealed a significant interaction with the background condition (Trial*Condition: χ^2^ = 19.3, df:3, *p* < 0.001), which was introduced by the *Optic Flow Condition.* In this case, bees decreased their speed at the end of the training, so that a significant difference was detected against the other background conditions (Trial*Condition: *‘Optic Flow’* vs. *‘Transparent’*: t = 3.64, *p* < 0.001, *Optic Flow* vs. *No Optic Flow*: t = 3.79, *p* < 0.001 and *‘Optic Flow’* vs. *‘Rotating Grating’*: t = 3.47, *p* < 0.001). This decrease was concomitant with an increase in the proportion of bees not choosing (Fig. 4B) so that it may reveal a reduction in motivation at the end of training in this background condition. The tortuosity of the trajectories (Fig. 5C) was neither affected by the succession of trials nor by the background condition (*Trial*: χ^2^ = 0.17, df:1, *p* = 0.68; *Condition*: χ^2^ = 3.62, df:3, *p* = 0.31), thus confirming that the structure of motor patterns was similar across the background conditions. Finally, the analysis of choice latency (Fig. 5D) showed a significant decrease along trials (*Trial*: χ^2^ = 21.85, df:1, *p* < 0.001; Fig. 5D), suggesting an improvement in the bee’s capacity to navigate in the VR environment. This evolution was independent of the background displayed (*Condition*: χ^2^ = 1.67, df:3, *p* = 0.65) but a tendency towards larger latencies was observed for the *‘Optic Flow Condition’.*

### Test performance

**Figure 6:**
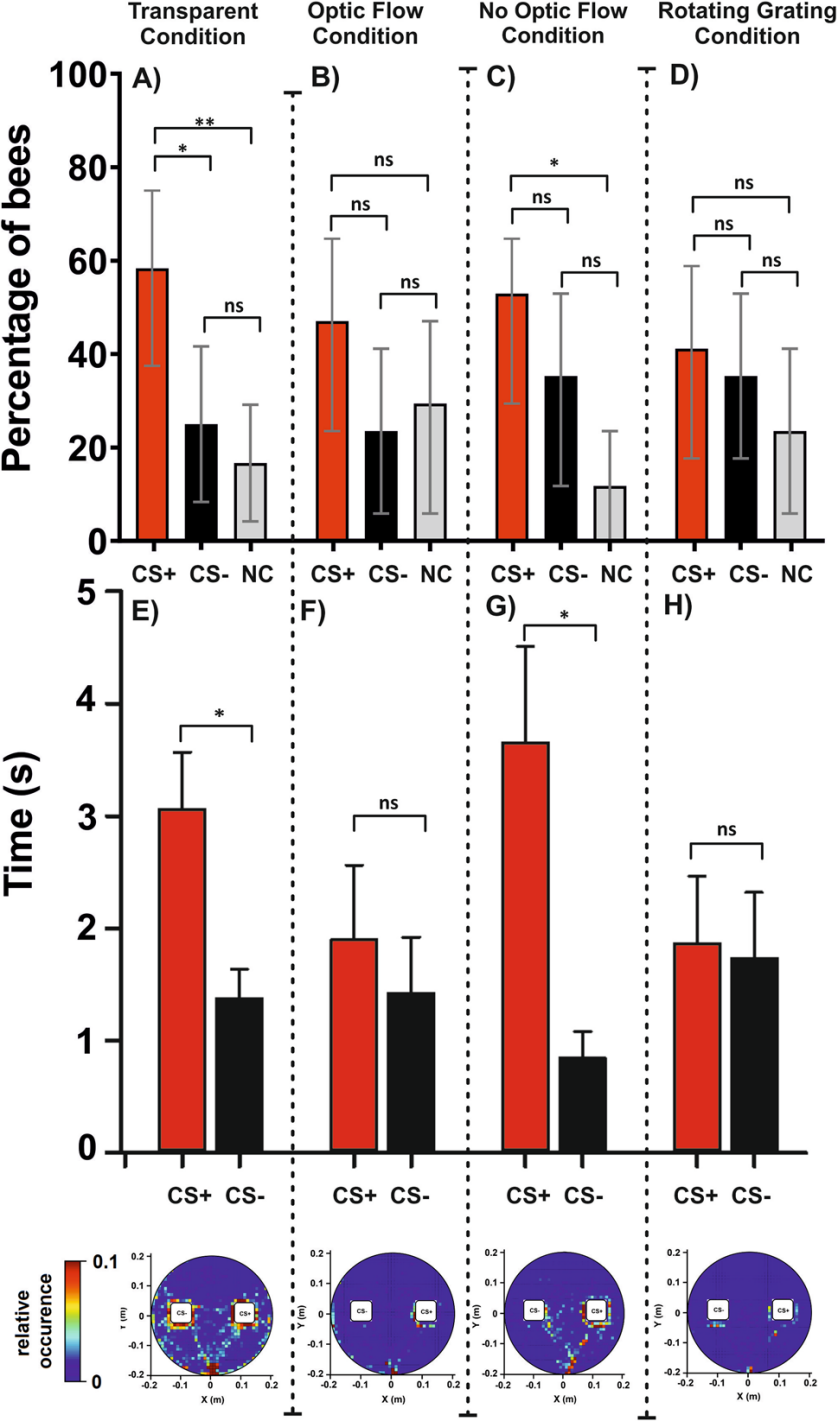
Test performances (1st choice and fixation time) in a color discrimination learning task under four different background conditions. Panels (**A**–**D)** refer to the 1st choice and show the percentage of bees responding to the CS+ (red), to the CS− (black) or not making any choice (NC; gray) during a retention test performed in extinction conditions after a 10-trial training. Error bars indicate 95% confidence intervals. *: *p* < 0.05; **: *p* < 0.01; ns: non-significant. (**A**) In the ‘*Transparent Condition*’ (N = 24), the blue and green cuboids were displayed against a dark background. (**B**) In the ‘*Vertical Grating—Optic Flow Condition*’ (N = 17), the cuboids were presented against a red-and-black vertical grating, which was coupled to the bee’s movements (closed-loop conditions). (**C**) In the ‘*Vertical Grating—No Optic Flow Condition*’ (N = 17), the cuboids were displayed against the same red-and-black grating but motion cues from the background were suppressed by keeping it constantly fixed to the bee’s gaze. (**D**) In the ‘*Rotating Vertical Grating Condition*’ (N = 17), the cuboids were shown against the same red-and-black grating, which was rotated counterclockwise around the virtual arena at a constant speed, thus generating a constant optic flow even when the bee did not move. Panels (**E**–**H**) refer to the fixation time, i.e. the time spent fixating either the CS+ or the CS− during the test. Bars represent the mean fixation time. Error bars indicate the standard error f the mean. *: *p* < 0.05; ns: non-significant. (**E**) ‘*Transparent Condition*’ (N = 24). (**F**) ‘*Vertical Grating—Optic Flow Condition*’ (N = 17). **G)** ‘*Vertical Grating—No Optic Flow Condition*’ (N = 17). (**H**) ‘*Rotating Vertical Grating Condition*’ (N = 17). The bottom row shows the heat map corresponding to each condition. Each heat map shows the cumulative coordinates occupied by the bees under each background condition during the test. Coordinates were binned into 1 cm^2^. Warmer colors depict locations more frequently occupied (see color bar). The highest frequency is cut down to 10% of the maximum on the color bar. The rewarded stimulus was placed arbitrarily on the right.

After the end of training, each bee was subjected to a test in which the green and the blue cuboids were presented in extinction conditions (no reinforcement provided). We recorded the percentage of bees choosing correctly the CS+ or the CS− in their first choice, or not choosing (NC) and the time spent fixating the CS+ and the CS− (Fig. 6).

The rewarded color did not affect the first choice during the test (*Color*: χ^2^ = 0, df:1, *p* = 1), so that performances could be analyzed irrespective of color identity within each background condition. Only under the *‘Transparent Condition’* (Fig. 6A), the difference between the percentages of CS+ and CS− responding bees was significant (CS+ vs CS− , z = 2.33, *p* = 0.02). The difference between the CS+ responding bees and the NC bees was also significant (CS+ vs. NC: z = 2.83, *p* = 0.005). On the contrary, no difference was detected between the CS− responding bees and the NC bees (CS− vs. NC: z = 0.71, *p* = 0.48). For the other three background conditions, no significant differences were detected between the percentage of bees choosing the CS+ or the CS− (Fig. 6B: *‘Optic Flow Condition’*; CS+ vs CS− , z = 1.41, *p* = 0.16; Fig. 6C: *‘No Optic Flow Condition’*; CS+ vs CS− , z = 1.0, *p* = 0.30; Fig. 6D: *‘Rotating Grating Condition’*; CS+ vs CS− , z = 0.4, *p* = 0.7). The comparisons with NC bees in these three conditions were all non-significant except in the *‘No Optic Flow Condition’* where CS+ responding bees and NC bees differed significantly (Fig. 6B: *‘Optic Flow Condition’*; CS+ vs. NC: z = 1.05, *p* = 0.30; CS− CS− vs. NC: z = 0.38, *p* = 0.70; Fig. 6C: *‘No Optic Flow Condition’*; CS+ vs. NC: z = 2.38, *p* = 0.02; CS− CS− vs. NC: z = 1.55, *p* = 0.12; Fig. 6D: *‘Rotating Grating Condition’*; CS+ vs. NC: z = − 1.09, *p* = 0.28; CS− vs. NC: z = − 0.75, *p* = 0.45). Overall, the first-choice data show that a significant discrimination between the CS+ and the CS− occurred in the *‘Transparent Condition’*, i.e. in the total absence of background information.

The analysis of the fixation time confirmed and extended this conclusion (Fig. 6E–H). Again, no discrimination learning was observed for the conditions in which motion cues were available from the background; bees spent the same amount of time fixating the CS+ and the CS− both in the *‘Optic Flow Condition’* (Fig. 6F; Wilcoxon U rank Test: V = 69, *p* = 0.63) and in the *‘Rotating Grating Condition’* (Fig. 6H; V = 83, *p* = 0.78). On the contrary, and consistent with the analysis based on the 1st choice, bees in the ‘*Transparent Condition*’ learned the discrimination between the CS+ and the CS− as they spent more time fixating the rewarded color than the non-rewarded one (Fig. 6E; V = 203, *p* = 0.049). Interestingly, a significant discrimination was also observed for the *‘No Optic Flow Condition’* (Fig. 6G; V = 128, *p* = 0.012)*,* a condition for which the 1st choice did not reveal significant differences. This result indicates that the reduction of motion cues inherent to the *‘No Optic Flow Condition’* also favored the occurrence of color learning, in agreement with what was observed for the *‘Transparent Condition’*.

The heat maps displaying the cumulative locations occupied by the bees’ trajectories during the entire test are shown in the bottom of Fig. 6. In these maps, the CS+ is displayed on the right by convention. In the *‘Transparent Condition’* (Fig. 6A), besides choosing significantly more the correct cuboid upon their first choice and spending more time fixating it, bees consistently walked towards the cuboids and inspected them. This tendency was not visible in the conditions in which motion cues were available from the background (Fig. 6B: *‘Optic Flow Condition’* and Fig. 6D: *‘Rotating Grating Condition’*), thus showing the impairment of performances induced by these cues. In the *‘No Optic Flow Condition’* (Fig. 6C), bees walked towards the cuboids and their choice was slightly biased towards the correct color, in accordance with the longer fixation time elicited by this color. Overall, these results reveal a negative influence of motion cues from the vertical background on visual-discrimination learning under VR conditions.

### Experiment 3: influence of ventral optic cues on visual discrimination learning

We assessed the importance of the ventral optic flow by training two different groups of bees to discriminate the green from the blue cuboid in the previous *‘Transparent Condition’* in which color learning was possible*.* The groups differed in the Styrofoam ball onto which the bees walked. For one group, the ball was homogenously white (Fig. 1C) so that no ventral motion cues were available to the walking bees (*‘No Ventral Optic Flow Condition’,* N = 29 bees). For the other group, the ball presented a black-and-white checkered pattern made of 7 mm^2^ squares (Fig. 1D; *‘Ventral Optic Flow Condition’*, N = 38 bees) so that ventral optic flow was available to the walking bees.

### Discrimination learning during training

**Figure 7:**
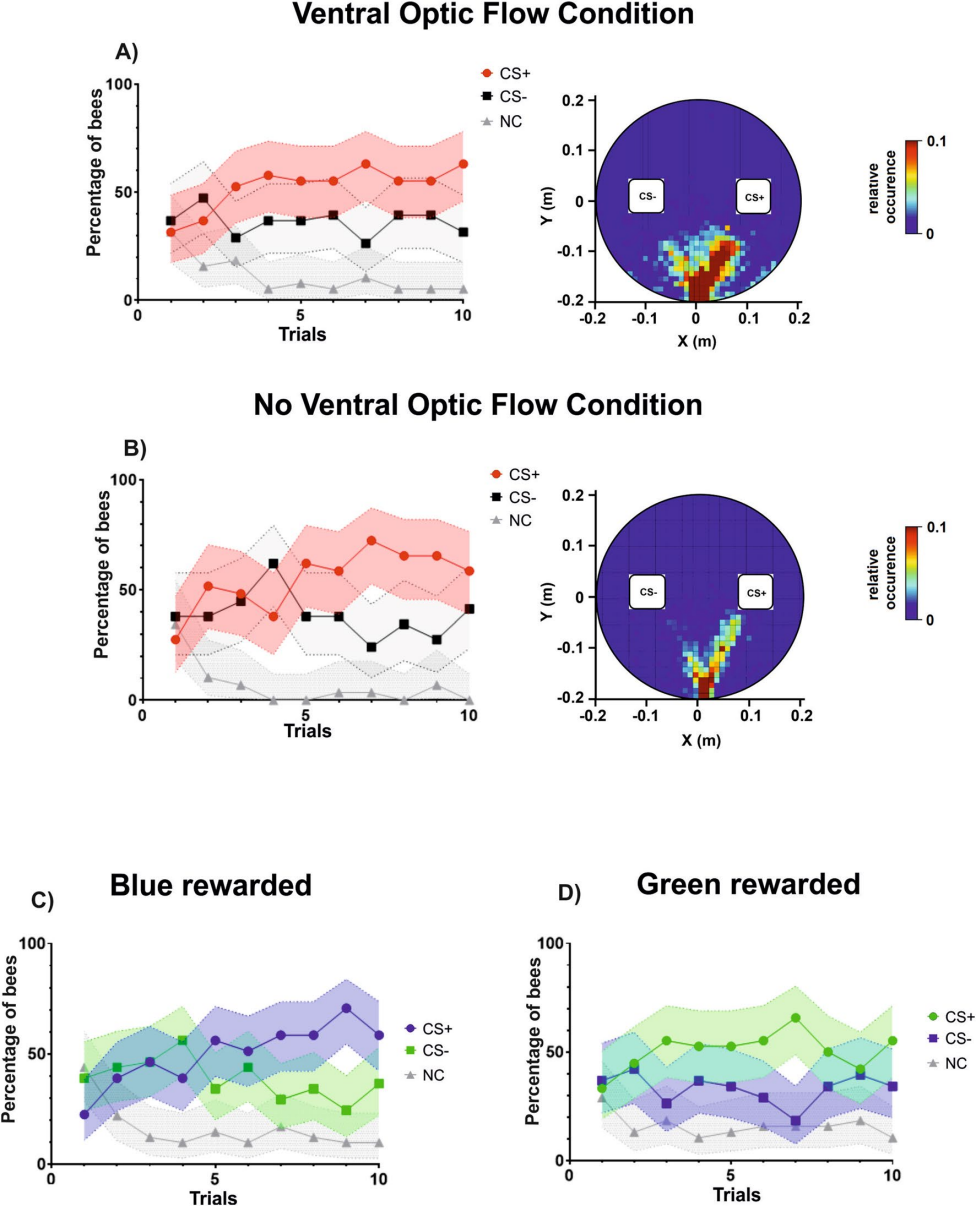
Acquisition performances in a color discrimination learning task under two different ventral optic-flow conditions. (**A**) Color discrimination learning with motion cues available ventrally on the treadmill (N = 38). Left: Acquisition curves in terms of the percentage of bees responding to the CS+ (red), to the CS− (black) or not making any choice (NC; gray) during the ten conditioning trials. The pink, light gray and gray areas around the curves represent the 95% confidence interval of CS+, CS− choices and NC, respectively. Right: Heat map showing the cumulative coordinates occupied by the bees trained under this condition during the ten training trials. Coordinates were binned into 1 cm^2^. Warmer colors depict locations more frequently occupied (see color bar). The highest frequency is cut down to 10% of the maximum on the color bar. While the side of the rewarded stimulus was randomized along conditioning trials, it was placed arbitrarily on the right in the heat maps. (**B**) Color discrimination learning in the absence of ventral motion cues on the treadmill (N = 29). Left: Acquisition curves as in (**A**). Right: Heat map as in (**A**). (**C**) Data pooled for the two optic-flow conditions and segregated according to the situation in which the CS+ color was Blue while the CS− color was Green. Acquisition curves in terms of the percentage of bees responding to the CS+ (blue), to the CS− (green) or not making any choice (NC; gray) during the ten conditioning trials. The blue, green and gray areas around the curves represent the 95% confidence interval of blue+, green− choices and NC, respectively. (**D**) Data pooled for the two optic-flow conditions and segregated according to the situation in which the CS+ color was green while the CS− color was blue. Acquisition curves in terms of the percentage of bees responding to the CS+ (green), to the CS− (blue) or not making any choice (NC; gray) during the ten conditioning trials. The green, blue and gray areas around the curves represent the 95% confidence interval of green+, blue− choices and NC, respectively.

Learning curves were again obtained by recording the percentage of bees correctly choosing the CS+ or the CS− in their first choice, or not choosing any stimulus (NC) during each trial. Figure 7A,B shows the learning curves obtained under the two ventral optic flow conditions and the cumulative heat map showing equal interaction with the two cuboid sides along trials. Yet, in this case the model that provided the best fit to the data included a three-way interaction between choices, trial number and color (Color*Trial*Choice: χ^2^ = 64.30, df:7, *p* < 0.001) but with no significant effect of the type of ball used (*Condition*: χ^2^ = 0, df:1, *p* = 1). This shows that the availability of ventral optic flow did not influence the bees’ performance when the variable quantified was the stimulus choice and that, on the contrary, a color effect existed. To analyze this effect, we pooled acquisition performances irrespective of the ventral optic flow condition, and represented them in terms of a green vs. blue discrimination (Fig. 7C: blue+ vs. green−; Fig. 7D: blue− vs. green +).

In both color conditions, the percentages of bees choosing the stimuli varied along trials (Choice*Trial: Blue: χ^2^ = 48.86, df:2, *p* < 0.001; Green: χ^2^ = 15.47, df:2, *p* < 0.001). However, the dynamic of the percentage of bees choosing the CS+ and that of bees choosing the CS− differed significantly only when blue was the rewarded color. In this case, the percentages of bees responding to blue (CS+) and to green (CS−) along trials differed significantly (Fig. 7C; Choice*Trial: CS+ vs. CS− : z = 4.88, *p* < 0.001). In addition, both the percentages of bees responding to the rewarded blue and to the punished green differed significantly from the non-responding bees along trials (CS+ vs. NC: z = 5.93, *p* < 0.001; CS− vs. NC: z = 2.62, *p* = 0.009). Segregating these data between the blue-rewarded bees that experienced the ventral optic flow condition and those that did not experience ventral optic flow yielded the same result. In both cases, the dynamic of the percentage of bees choosing the blue+ and that of bees choosing green- differed significantly (*‘Ventral Optic Flow’*: CS+ vs. CS− : z = 3.62, *p* < 0.001; *‘No Ventral Optic Flow’*: CS+ vs. CS− : z = 3.31, *p* < 0.001). When green was the rewarded color, no significant differences in the percentages of bees responding to green+ and that of bees responding to blue – was detected along trials, even if the former tended to be higher than the latter (Fig. 7D; Choice*Trial: CS+ vs. CS− : z = 0.60, *p* = 0.55). Both percentages were significantly higher than that of bees not responding to any stimulus (CS+ vs. NC: z = 3.58, *p* < 0.001; CS− vs. NC: z = 3.23, *p* = 0.001). The same pattern of responses with respect to bees responding to green+ and to blue- was found when analyzing separately the two optic-flow conditions (*‘Ventral Optic Flow’*: CS+ vs. CS− : z = 0.23, *p* = 0.82; *‘No Ventral Optic Flow’*: CS+ vs. CS− : z = 1.16, *p* = 0.25).

### Motor and temporal components of bee trajectories during training

**Figure 8:**
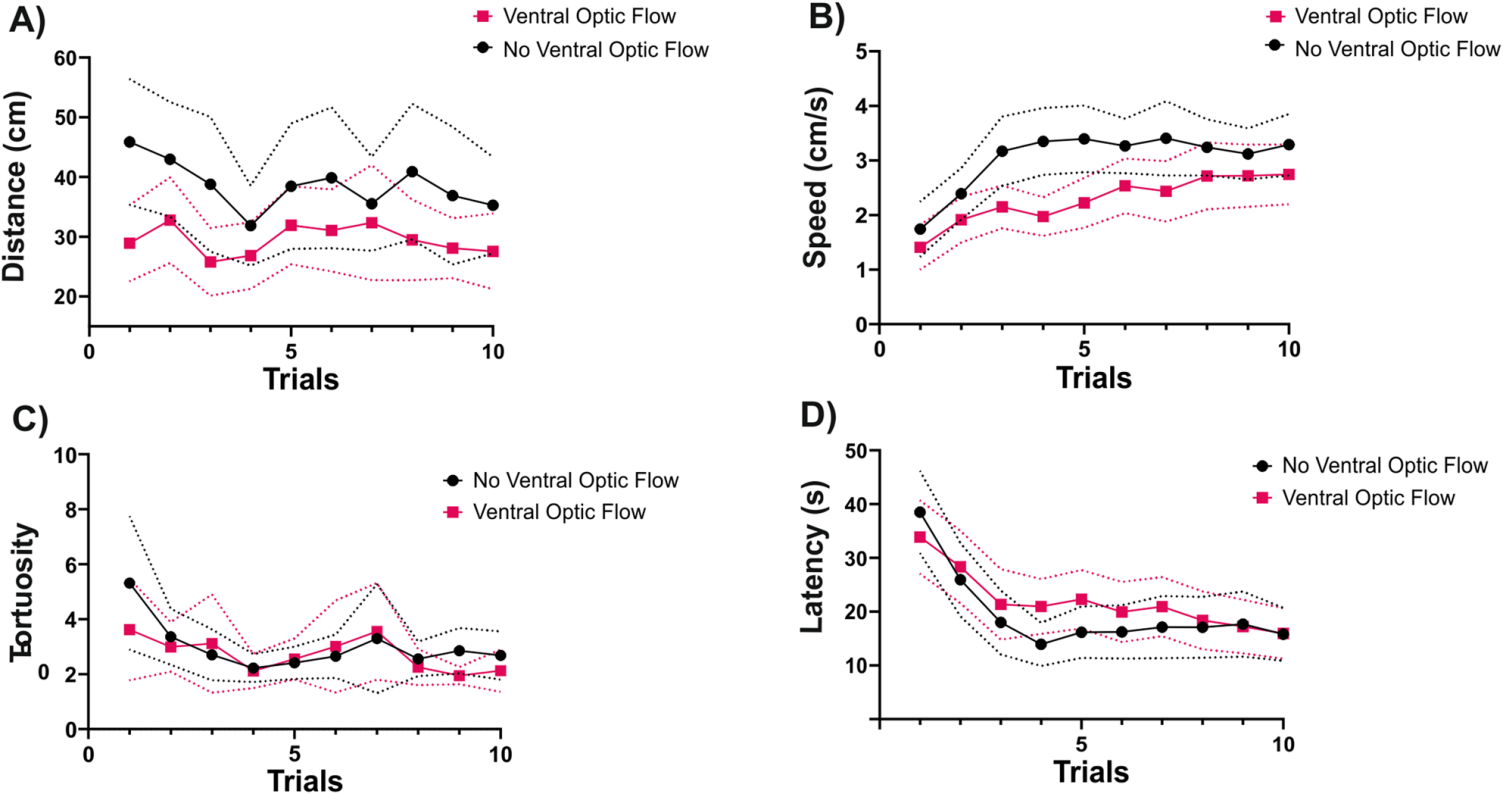
Motor and temporal components of bee trajectories during the acquisition trials. For each ventral optic-flow condition, the evolution of (**A**) the distance walked, (**B**) the walking speed, (**C**) the tortuosity and (**D**) the choice latency during training trials is shown. The tortuosity was the ratio between the total distance walked and the straight line connecting the first and the last point of the trajectory during a training trial. ‘*Ventral Optic Flow’* (N = 38), ‘*No Ventral Optic Flow*’ (N = 29). The dashed lines above and below the curves represent the 95% confidence interval.

We analyzed the motor performance of bees in the two conditions described above to determine if and how ventral motion cues affected the displacement of bees in the VR setup during the training trials (Fig. 8A–D). The distance walked during the acquisition phase (Fig. 8A) was affected by the presence of ventral optic flow (*Condition*: χ^2^ = 7.45, df:1, *p* = 0.006). With the checkered ball, the bees walked less. The walking speed during the acquisition phase (Fig. 8B) was also significantly slower when ventral optic flow was available (*Condition*: χ^2^ = 6.03, df:1, *p* = 0. 01) although it increased significantly over trials for both conditions (*Trial*: χ^2^ = 85.20; df: 1, *p* < 0.0001). The tortuosity of the walking paths (Fig. 8C) decreased over trials (*Trial*: χ^2^ = 7.95, df: 1, *p* = 0.005) but was unaffected by the ventral optic flow (*Condition*: χ^2^ = 0.56, df:1, *p* = 0.45). Finally, the latency before making a choice (Fig. 8D) was stable over trials even if an apparent decrease was observed in the first trials (*Trial*: χ^2^ = 1.97; df: 1, *p* = 0.16), and was not influenced by the ventral optic flow (*Condition*: χ^2^ = 0.19, df:1, *p* = 0.66). Overall, the significant variation in distance walked and walking speed detected between the two conditions shows that bees were not insensitive to the presence of ventral motion cues. They perceived them and in consequence walked slower and less.

### Test performance

**Figure 9:**
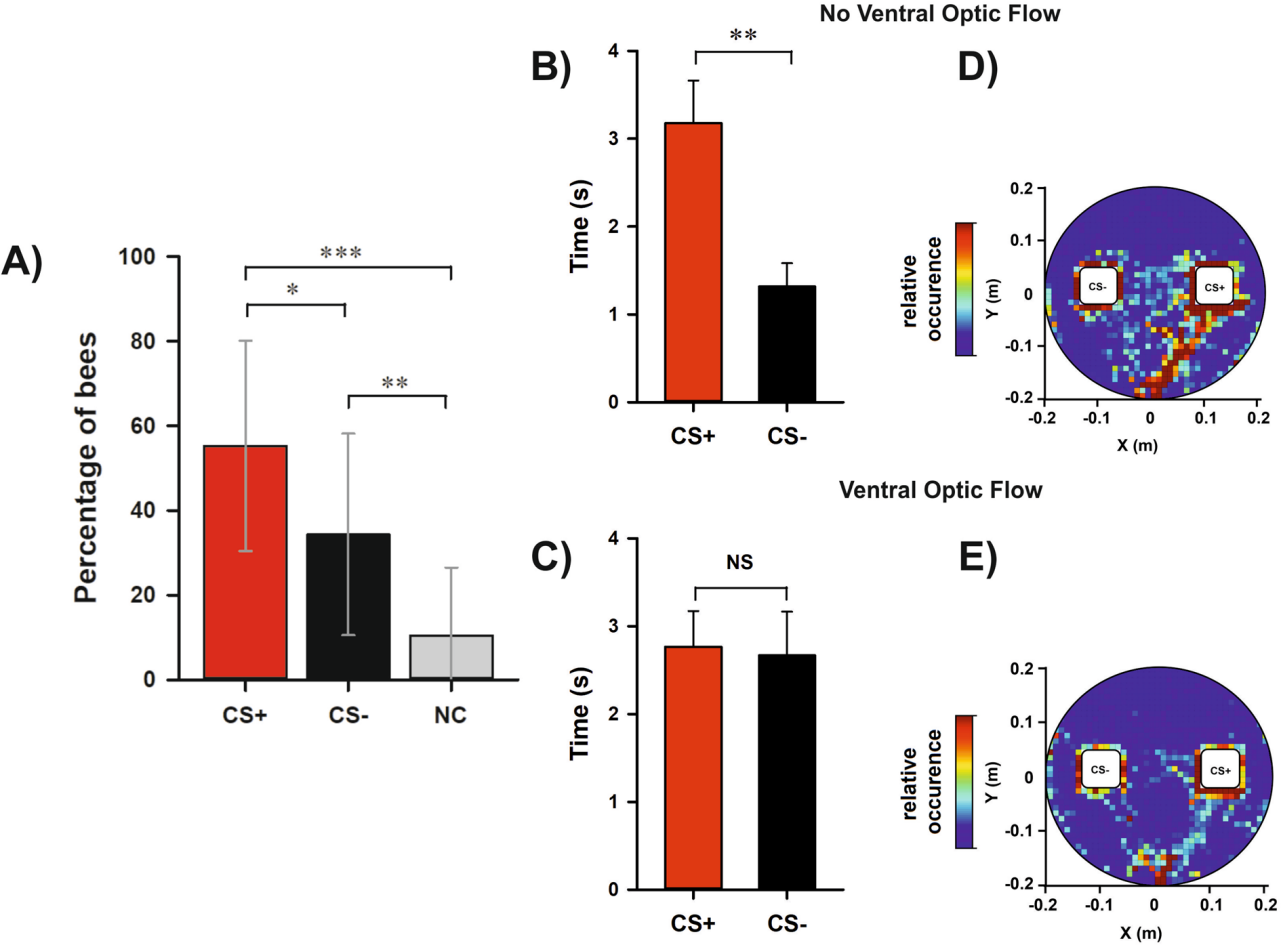
Test performances (1st choice and fixation time) in a color discrimination learning task under two different ventral optic-flow conditions (with ventral optic flow and without ventral optic flow). (**A**) 1st choice performed during the test. As there were neither significant differences between the two ventral optic-flow conditions nor between the color conditions (blue or green rewarded), results were pooled and presented as a single bar diagram (N = 67). The graph shows the percentage of bees responding to the CS+ (red), to the CS− (black) or not making any choice (NC; gray) during the retention test. Error bars indicate 95% confidence intervals. *: *p* < 0.05; **: *p* < 0.01; ***: *p* < 0.001. (**B**, **C**) Fixation time during the test in the ‘*No Ventral Optic Flow Condition*’ and in the ‘*Ventral Optic Flow Condition*’, respectively. In this case, fixation times were separated according to the experimental condition, as different response patterns were observed with and without ventral optic flow. The graphs show the mean time (± S.E.) spent fixating either the CS+ or the CS− during the retention test. (**B**) In the ‘*No Ventral Optic Flow Condition*’, bees fixated significantly longer the CS+ than the CS−. **: *p* < 0.01. (**C**) In the ‘*Ventral Optic Flow Condition*’, bees fixated equally the CS+ and the CS−. NS: not significant. (**D**, **E**) Heat maps showing the cumulative coordinates occupied by the bees during the test in the ‘*No Ventral Optic Flow Condition*’ and in the ‘*Ventral Optic Flow Condition*’, respectively. The CS+ is shown on the right by convention. Coordinates were binned into 1 cm^2^. Warmer colors depict locations more frequently occupied (see color bar). The highest frequency is cut down to 10% of the maximum on the color bar. (**D**) In the ‘*No Ventral Optic Flow Condition*’, bees clearly aimed at the CS+ besides choosing it more frequently in their first choice. (**E**) In the ‘*Ventral Optic Flow Condition*’, bees also aimed at the CS+ but in a less clear way.

After the end of training, each bee was subjected to a test in which the green and the blue cuboids were presented in extinction conditions (no reinforcement provided) in the presence or absence of ventral optic flow. We recorded the percentage of bees correctly choosing the CS+, the CS− or not choosing (NC). There was no significant effect of the ventral optic flow on test performances when the variable considered was the choice made by the bees (*Condition*: χ^2^ = 0, df:1, *p* = 1). Thus, the results of both groups of bees were pooled (N = 67) and shown as a single graph (Fig. 9A). In this case, the color of the CS+ did not affect the performance (*Color*: χ^2^ = 0, df:1, *p* = 1), thus showing that the color effect detected during training was not consistent. In the test, bees preferred the CS+ over all conditions (CS+ vs. CS− : z = 2.41, *p* = 0.02; CS+ vs. NC: z = 5.03, *p* < 0.0001; CS− vs. NC: z = 3.16, *p* = 0.002), thus confirming that they had learned the color discrimination during acquisition.

The analysis of the fixation time showed a significant difference between the conditions *‘Ventral Optic Flow’* and *‘No Ventral Optic Flow’.* Bees fixated significantly longer the CS+ than the CS− in the absence of ventral optic flow (Fig. 9B; V = 320, *p* = 0.008) while they fixated equally both stimuli in the presence of ventral optic flow (Fig. 9C; V = 373; *p* = 0.75). The first condition is identical to the ‘*Transparent Condition*’ previously studied, in which a white ball was used as a treadmill. The results were, therefore, consistent between the two experiments: bees preferred the CS+ in their first choice and spent more time fixating it. The second condition shows that ventral motion cues played a distractive role as only when they were absent, did the fixation time correlate with the bees’ choice.

The heat maps displaying the cumulative locations occupied by the bees’ trajectories during the entire test are shown in Fig. 9D,E. In the *‘No Ventral Optic Flow’* condition (Fig. 9D), bees consistently walked towards the CS+ cuboid and inspected it during the test besides choosing it more frequently upon their first choice. In the *‘Ventral Optic Flow’* condition (Fig. 9E), bees still walked towards the CS+ cuboid but in a less clear way.

## Discussion

We studied the impact of motion cues provided by the background on visual-discrimination learning by honey bees in virtual-reality conditions. Bees had to learn the difference between two virtual color cuboids, one of which was rewarded while the other was punished. We focused both on motion cues derived from a background placed frontally ‘behind’ the color stimuli, and from a ventral ground, which was perceived in the ventral visual field while the bee walked on a Styrofoam ball. In the latter case, the perceived optic flow had no direct relation with the cuboids perceived in the frontal field. The color discrimination task was set under closed loop conditions so that in the case of the grating displayed frontally, both the cuboids and the background could vary (translation and expansion) according to the bees’ movements. Our results indicate that in VR conditions, frontal but no ventral motion cues from the background interfered with the learning of colors. Although ventral motion cues did not affect color learning, they were well perceived as they affected walking distance and speed and impaired fixation time of the rewarded stimulus during the test.

### Optic flow and visual performances in insects

Optic flow is the pattern of apparent motion of objects, surfaces, and edges in a visual scene caused by the relative motion between an observer and a scene^40,41^. It can be seen as a vector field that gives the retinal slip speed of each contrasting object encountered in the environment when the observer moves and/or when features in the environment move relative to the observer^42^. Optic flow processing is crucial for navigation as it allows assessing the distance to objects encountered. Objects closer to an observer move faster in the retinal field than distant objects, so that approaching a target induces higher optic flow while moving away from it decreases it. This information is crucial for moving insects as it allows estimating distances in translational segments^43–46^ and avoiding collisions with circumventing obstacles and flying equidistantly from parallel landmarks. For instance, when flying along narrow corridors, insects use the magnitude of visual motion experienced in each eye to control their position, height and speed^47–49^.

Motion cues can be extracted at the edge of objects through parallax and allow evaluating the distance of targets with respect to their background based on differences in their relative retinal speed^50–54^. Edges are therefore contrasting regions in terms of motion-parallax cues and are privileged by flying insects in their detection and landing strategies^51^. Numerous experiments have documented this fact in honey bees^50–54^. An interesting example is provided by experiments in which bees were trained to solve a discrimination between a plain black disk and a black ring positioned a few centimeters in front of a white background. The targets provided a good contrast to the background both in terms of intensity as well as in terms of the motion cues provided at their edges so that bees had no problems in learning this shape discrimination^55^. However, when bees were trained on the same shapes, yet cut from a textured paper and placed in front of a similarly textured background, the task was impossible for them^55^. This result shows that motion cues alone, which existed because the textured targets were placed in front of the textured background, are not always helpful to appreciate shape differences between targets. Interestingly, this impossible discrimination became possible after the bees were primed by pre-training them with the easy discrimination involving plain stimuli against the white background. This improvement shows that attentional mechanisms boosted by the priming procedure are crucial for achieving target/background segmentation. The role of attentional mechanisms will be discussed below.

### Ventral motion cues did not influence the color discrimination performance of bees in VR but affected walking parameters and fixation time

Multiple layers of neurons within visual circuits in the bee brain are devoted to the segregated processing of motion cues, which are essential to estimate distances traveled in translational pathways^56^. Ventral optic flow is particularly important for insects flying in open spaces. In consequence, flying above surfaces providing strong optic flow cues is preferred by bumble bees over flying above featureless backgrounds^57^. Experiments on bumblebees trained to fly along textured tunnels showed that in tunnels of 60 and 120 cm width, control of the lateral position was achieved by balancing the magnitude of translational optic flow experienced in the lateral visual field of each eye; yet, in wider tunnels, bumblebees used translational optic flow perceived in the ventral visual field to control their lateral position and to steer along straight tracks^57^. Ventral optic flow can be used to keep a constant height above the ground using a feedback control loop in which a set point value of perceived ventral optic flow is maintained constant by varying the lift, a solution that was shown experimentally in flying bees^58,59^ and that proved to be efficient when implemented in flying robots that needed to keep a constant fly height^60^.

These and other findings^59,61–63^ clearly show the importance of ventral motion cues for the translational displacements of flying insects. Although less information is available for walking insects, experiments performed on desert ants *Cataglyphis fortis* walking in narrow tunnels showed that both the lateral and the ventral optic flow were dispensable for distance estimation^64^. In these insects, the use of a ‘pedometer’ was proposed, i.e. a stride integrator that accounts for stride number and the respective stride length^65,66^. Although optic flow can be computed by these ants, as shown by the case of ants transported by nestmates, which rely on the optic flow perceived during their transport^67^, the primary mechanism to gauge distances is based on idiothetic cues.

In our experiments, bees walking on a Styrofoam ball were partially affected by the presence or absence of ventral optic-flow cues (Figs. 7, 8, 9). During the training, these cues did not affect the learning performance measured in terms of color choice (Fig. 7). Yet, we found an effect of color, suggesting that discrimination learning was better when blue was the rewarded color. However, this effect disappeared during the test (Fig. 9), as the first choice of the bees revealed that they preferred significantly the CS+, irrespective of its color. Ventral motion cues affected the other variable recorded during the test, the time spent fixating the cuboids (Fig. 9B,C). When these cues were absent, bees fixated more the CS+, consistently with their first color choice; however, when ventral motion cues were available, they fixated both the CS+ and the CS− to similar extents, even if they preferred the CS+ in their first color choice. Thus, ventral motion cues interfered with the time spent fixating the CS+ during the test.

The absence of effect of ventral motion cues during the training did not mean that bees did not pay attention to them or that they were unable to perceive the difference between the two walking surfaces. Figure 8A,B shows that both the distance walked and the walking speed decreased significantly when ventral motion cues were available, thus showing that bees perceived them. Their impact on these motor variables indicates, in addition, that such cues are relevant for estimating walking distances. This conclusion goes against the possibility that in a walking context, bees, like desert ants, rely on a mechanism for estimating distances different from that employed during flight. In fact, it is difficult to conceive how the relevance of optic flow could be switched off during walking, given its fundamental role for bee navigation.

Alternatively, our findings may indicate that ventral optic-flow cues play a fundamental role *en route* to the goal for distance estimation and completion of an intended translational vector, but not in the immediate vicinity of the goal, when the insect faces the task of close-up object recognition. In the latter situation, translational ventral optic flow may be irrelevant as the goal has been reached. Last but not least, it is worth considering that our experiments did not create a ventral optic flow in the virtual arena, i.e. below the targets to be discriminated, but only on the walking treadmill. Including ventral motion cues in the floor of the virtual arena could affect the choice of the color cuboids in a way similar to that induced by the frontal motion cues from the background.

### Frontal motion cues from the background interfered with the color discrimination performance of bees in VR

Motion cues perceived frontally at the edges of vertically displayed targets allows segregating them from their respective background based on motion parallax cues. This feature extraction improves therefore object identification and landing on targets. Yet, in our experiments, whenever motion cues from the background were available (‘*Vertical Grating—Optic Flow Condition*’ and ‘*Rotating Vertical Grating Condition*’; see Fig. 4), color discrimination of objects located in the virtual foreground was impaired. This result is in contradiction with the hypothesis that animals should behave better in more realistic environments and challenges a priori efforts towards enriching our VR environment with additional cues besides those to be learned and discriminated. Indeed, learning was only possible in the total absence of a frontal background (‘*Transparent Condition’*, Fig. 4), suggesting that background cues interfered with the learning of foreground objects. Interestingly, in the ‘*Vertical Grating—No Optic Flow Condition*’, optic flow from the background was artificially suppressed and yet learning was not apparent even if a tendency towards a segregation of CS+ and CS− curves was observed (see Fig. 4B). However, when test performances were analyzed in terms of the time spent fixating the CS+ and the CS− , a significant difference in favor of the former was found, which is consistent with a learning effect. The fact that performances were not as clear as in the *‘Transparent Condition’* suggests that the mere presence of the background may have been distractive for the bees. Thus, both the motion cues emanating from the background, and its illumination conditions, may have interfered with color learning in the VR arena.

A first explanation for this interference could rely on the role of irradiance cues used to establish the background in our VR environment. The background was projected onto the semicircular screen of our setup by a videoprojector and therefore provided irradiance cues that could have attracted the bees based on positive phototaxis, thus interfering with color discrimination. As bees are tested in the dark, a situation inherent to the use of a videoprojector, phototaxis may have indeed influenced the behavior of the bees in our VR setup as shown in experiments performed in open-loop conditions using the same kind of videoprojector-based display^12^. Admittedly, the green and the blue lights used for training the bees had 22 times and 143 times more irradiance than the red used for the background (Red 100: RGB: 255, 0, 0; irradiance: 1130 μW/cm^2^). It could be interesting to determine if a similar interference with color learning would take place when using the other red light that the bees could see (Red 50: RGB: 128, 0, 0; irradiance: 140 μW/cm^2^; see Fig. 3). For this light, the difference of irradiance between colors and background decreases in one order of magnitude with respect to the Red 100 used in our experiments. In theory, using the Red 50 light should not change the main findings reported because the phototactic attraction exerted by this stimulus was identical to that induced by the Red 100 light (see Fig. 3B,C).

Another reason for the negative influence of motion cues emanating from the frontal background could be an excessive salience of these cues with respect to those from the foreground objects that had to be discriminated. In natural conditions, background objects provide motion cues that are less salient than those of foreground objects. Although we attempted to reproduce this situation in our VR environment (the expansion of the cuboids during forward motion was 1.7°/cm while that of a red bar from the background was 0.18°/cm), the optic flow generated by the background might still have been too high and detracted bees from efficiently learning the discrimination task.

Finally, in the ‘*Rotating Vertical Grating Condition*’, optomotor responses triggered by the background rotating regularly in front of the bees may have interfered with the color learning task. To determine if this was the case, we analyzed the cumulative turning exhibited by the bees in this condition and in the ‘*Transparent Condition*’, where no background was available. Figure S2 shows that the cumulative turning in the direction of the rotating background (to the left) was significantly higher in the ‘*Rotating Vertical Grating Condition*’*,* which indicates the presence of optomotor responses. These responses may have interfered with color learning and may be one of the causes of the impaired performance observed.

### Chromatic and achromatic vision in the VR setup

Bees were trained to discriminate two different colors against an achromatic background. Blue and green colors differing in intensity were used to this end (Fig. S1A), which may have resulted in bees using differences in intensity rather than chromatic differences to solve the task when motion cues from the background did not interfere (i.e. in the *‘Transparent Condition’*; see Fig. 4A). Yet, this possibility is ruled out by the performance of the bees itself. In this condition, no asymmetries in color learning were observed depending on which color was rewarded. Had the bees been guided by achromatic intensity, then significant learning asymmetries should have emerged: bees trained to the less intense green should show impaired learning, detracted by the highly intense blue displayed by the alternative non-rewarded stimulus. On the contrary bees trained to the highly intense blue should have their performances amplified by the attraction induced by the blue light. This was not the case and no color effect was observed in this experiment. The situation presented in the *‘Transparent Condition’* was reproduced in the experiments studying the effect of the ventral optic flow, when the surface of the treadmill was plain white (see Fig. 7, *‘No Optic Flow Condition’*). In this case, a color effect consistent with the use of intensity was visible during the training, as performance was better when blue was rewarded than when green was rewarded (see Fig. 7C,D). However, this effect disappeared during the test (see Fig. 9), showing that it was inconsistent and that even the bees rewarded on the green color learned the task. These results indicate that in the absence of distractive motion cues from the background, the bees were mainly guided by chromatic cues from the blue and green colors although we cannot definitely rule out an incidence of stimulus intensity in these experiments. Note that the same colors were used in previous studies performed in our VR setup and that no color asymmetry was found, which goes against the use of color intensity by the bees^12–16,27^.

An additional issue that requires consideration is the possible interference of the red light (Red 100) used for the background with the color vision system of the bees involved in the blue-green discrimination. Figure S1 shows that Red 100 could only be perceived via the L (Green) receptor type, i.e. via an achromatic visual mechanism involving a single receptor type. Bees can see red (see Fig. 3), not as a color, but as an achromatic stimulus, perceived in terms of its intensity by the L receptor^32,33^. Whether Red 100 affected negatively chromatic discrimination, interfering with the L-receptor type involved in this discrimination is unknown. VR experiments with freely flying bumblebees trained to land on a virtual horizontal blue target located on a projected achromatic checkerboard made of random pink (RGB: 255, 127, 127) and white (RGB: 255, 255, 255) squares showed that the background had no incidence on the bees’ performance^61^. In this case, the pink light used could potentially stimulate all receptor types and thus truly affect the color vision system, contrarily to our red light (RGB: 255, 0, 0). The fact that this was not the case suggests a minor effect, if any, of the red light in our color discrimination experiments.

## Conclusion

Our results point towards deficits in attentional processes underlying color discrimination whenever motion cues from the background were frontally available in our VR setup. In the case of ventral motion cues, no interference of color learning was observed, yet, a distractive effect on the time spent fixating the stimuli was detected during the test. Attention plays a fundamental role in visual discrimination tasks achieved by bees and other insects^68–71^. Attention is defined as the “ability to focus our perception on one stimulus (or group of related stimuli), while filtering out other simultaneous stimuli that are less relevant at any moment”^72^. Several studies focusing on color discrimination by bees have underlined the importance of attention in this context. In particular, differential conditioning protocols—as the one used in this work—are said to require more attention than absolute conditioning, the simple training of a single stimulus^73^, in particular when the stimuli to be discriminated are similar^34^. The role of attention in visual object recognition was studied by training bees to choose a colored target disc among a variable number of differently colored distractor discs^74^. Accuracy and decision time were measured as a function of distractor number and color. For all color combinations, decision time increased and accuracy decreased with increasing distractor number, whereas performance improved when more targets were present.

From this perspective, highly salient irradiance or motion cues from the background may have interfered with attentional processes required to achieve the color cuboid discrimination. Further experiments may explore strategies to reduce their salience and thus enable their perceptual filtering in our VR landscape.

## Supplementary Information

Supplementary Information.
