# Supplementary material for: Motion cues from the background influence associative color learning of honey bees in a virtual-reality scenario
Source: Sci Rep. 2021 Oct 26;11:21127. doi: 10.1038/s41598-021-00630-x (PMC8548521; doi:10.1038/s41598-021-00630-x)
Supplement: Supplementary file 1 — Supplementary Information. [file 41598_2021_630_MOESM1_ESM.docx]

# **Supplementary Information**

# **Motion cues from the background influence associative color learning of honey bees in a virtual-reality scenario**

**Gregory Lafon^1^, Scarlett R. Howard^1¥^, Benjamin H. Paffhausen^1^, Aurore Avarguès-Weber^1^* and Martin Giurfa^1, 2, 3^***

**^1^** Research Centre on Animal Cognition, Center for Integrative Biology, CNRS (UMR 5169), University of Toulouse, 118 route de Narbonne, F-31062 Toulouse cedex 09, France

**^2^** College of Animal Sciences (College of Bee Science), Fujian Agriculture and Forestry University, Fuzhou 350002, China

**^3^** Institut Universitaire de France (IUF), Paris, France

**^¥^**: *Present address:* School of Life & Environmental Sciences, Melbourne Burwood Campus, Deakin University, Melbourne, VIC, Australia

^*^Senior authorship shared


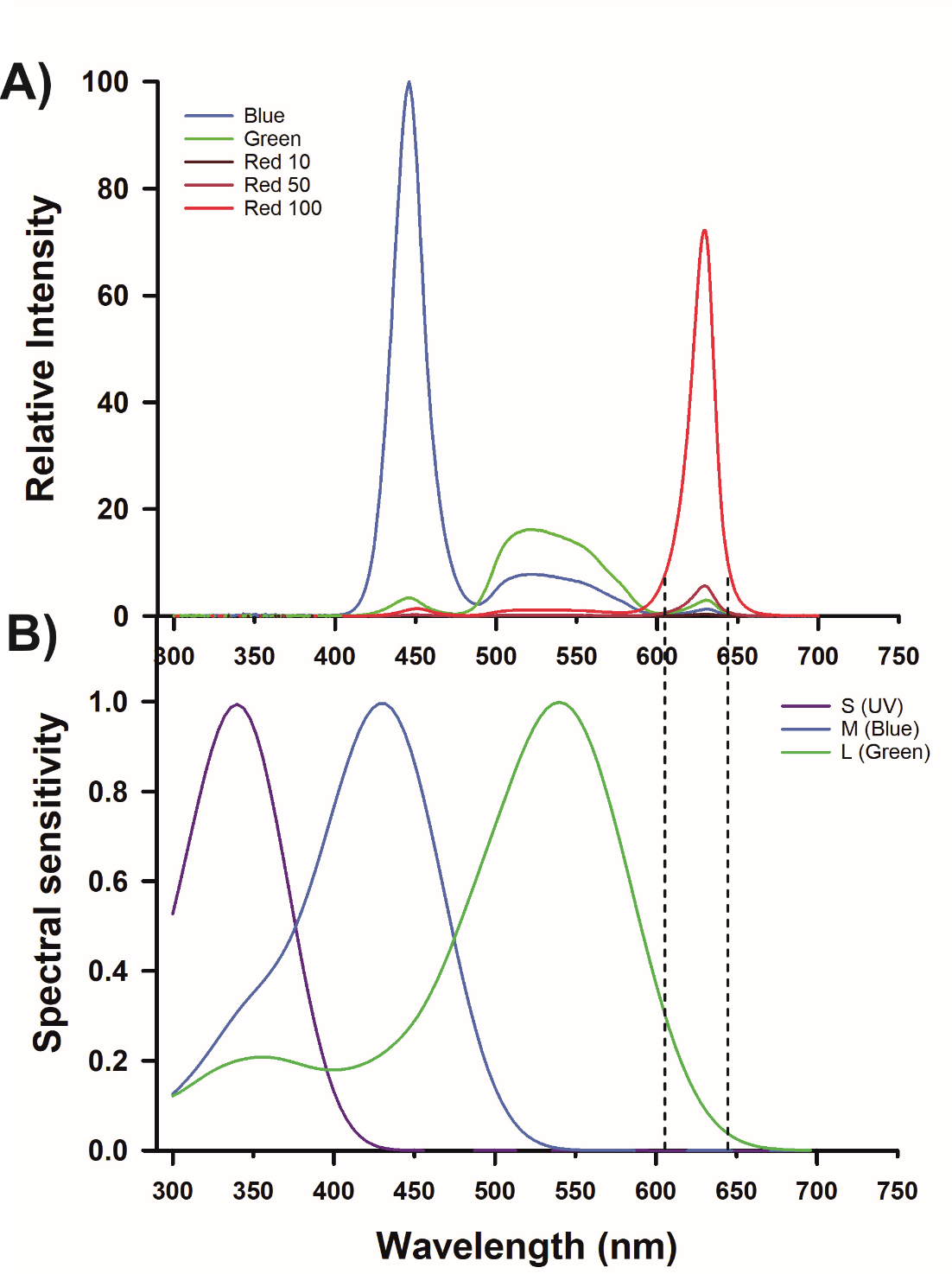


**Figure S1: Spectral emittance of the stimuli produced by the video projector and spectral sensitivity curves of the honey bee photoreceptor types. A)** Spectral distribution (relative intensity as a function of wavelength) of the blue light (dominant wavelength 446 nm) and the green light (dominant wavelength 528 nm) used to train the bees in the color discrimination task. The graph also shows the spectral distribution of the three types of red light tested (Red 10, Red 50 and Red 100). **B)** Spectral sensitivity curves of the three types of photoreceptors of the honey bee: S (short-wave or UV), M (mid-wave or blue) and L (long-wave or green). The vertical dashed lines indicate the region in which the red lights could potentially stimulate the L-receptor type.

**
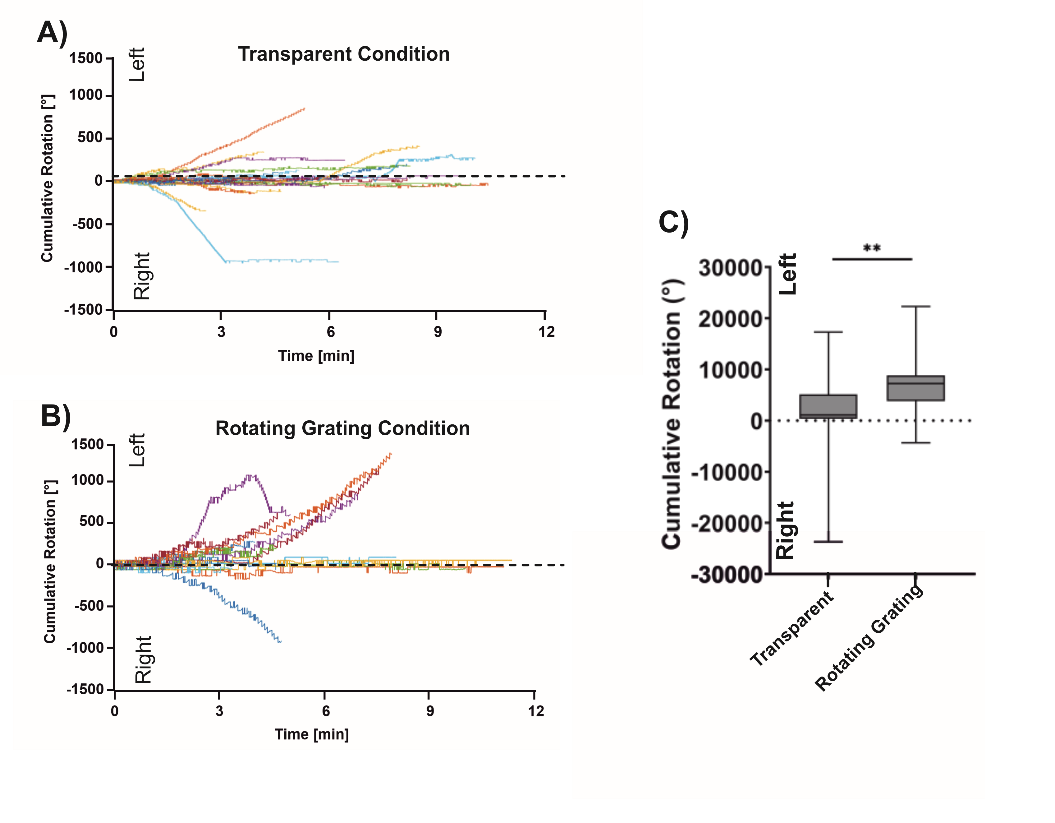
Figure S2: Comparison of turning behavior in the *Transparent Condition* and the *Rotating Grating Condition*.** A cumulative rotation score (°) was computed for every bee by adding 360° anytime the bee's heading changed from 0° to 360°, and -360° anytime it changed from 360° to 0°. Positive scores indicate left turning (counter clock-wise rotation) while negative values indicate right turning (clock-wise rotation). Individual trajectories are shown in different colors. **A)** Cumulative rotation score (°) of bees in the *Transparent Condition* (n = 24). In this condition, the green and blue cuboids were displayed against a static black background. The bees walked mainly forwards except for two individuals, blue and red, that exhibited a tendency to turn to the right and to the left, respectively. **B)** Cumulative rotation score (°) of bees in the *Rotating Grating Condition* (n = 17). In this condition, the green and blue cuboids were displayed against a black and red grating that rotated *to the left* (anti-clockwise) at a constant speed (12 m/s), thus generating an optic flow that was independent of the bee’s movements. A significant proportion of the bees turned to the left, thus following the rotating background, consistently with an optomotor response. **C)** Cumulative turning behavior (median, quartiles) computed across bees in the two conditions **A)** and **B)**. Bees in the *Rotating Grating Condition* turned more to the left, i.e. following the background grating, than in the *Transparent Condition* (Wilcoxon U rank test; W = 314.5, p = 0.00358). **: P < 0.01.

**Table S1**: GLMM Analysis Experiment 1 – Models comparison.

| Models | df | AIC | Log-Lik | χ^2^ | p(>χ^2^) |
| --- | --- | --- | --- | --- | --- |
| Model 1: Score ~ Choice*Intensity*Test + (1\|BeeID) | 19 | 654.5 | -308.2 |  |  |
| Model 2: Score ~ Choice*Intensity+Test + (1\|BeeID) | 11 | 648.1 | -313.0 | 9.54 | 0.30 |
| Model 3: Score ~ Choice*Intensity + (1\|BeeID) | 10 | 646.1 | -313.0 | 0.002 | 0.97 |
| Model 4: Score ~ Choice+Intensity + (1\|BeeID) | 6 | 703.5 | -345.8 | 65.48 | <0.001*** |

**Table S2**: GLMM Analysis Experiment 2 – Models comparison.

| Models – *Acquisition Phase – Transparent condition* | df | AIC | Log-Lik | χ^2^ | p(>χ^2^) |
| --- | --- | --- | --- | --- | --- |
| Model 1: Score ~ Choice*Trial + (1\|BeeID) | 7 | 824.9 | -405.4 |  |  |
| Model 2: Score ~ Choice + Trial + (1\|BeeID) | 5 | 828.8 | -409.4 | 7.99 | 0.018* |

| Models – *Acquisition Phase – Vertical grating – No Optic Flow condition* | df | AIC | Log-Lik | χ^2^ | p(>χ^2^) |
| --- | --- | --- | --- | --- | --- |
| Model 1: Score ~ Choice*Trial + (1\|BeeID) | 7 | 571.9 | -278.9 |  |  |
| Model 2: Score ~ Choice + Trial + (1\|BeeID) | 5 | 581.4 | -285.7 | 13.58 | 0.0011** |

| Models – *Acquisition Phase – Vertical grating – Optic Flow condition* | df | AIC | Log-Lik | χ^2^ | p(>χ^2^) |
| --- | --- | --- | --- | --- | --- |
| Model 1: Score ~ Choice*Trial + (1\|BeeID) | 7 | 630.2 | -308.1 |  |  |
| Model 2: Score ~ Choice + Trial + (1\|BeeID) | 5 | 631.3 | -310.7 | 5.16 | 0.08 |

| Models – *Acquisition Phase – Rotating Vertical grating condition* | df | AIC | Log-Lik | χ^2^ | p(>χ^2^) |
| --- | --- | --- | --- | --- | --- |
| Model 1: Score ~ Choice*Trial + (1\|BeeID) | 7 | 617.1 | -301.5 |  |  |
| Model 2: Score ~ Choice + Trial + (1\|BeeID) | 5 | 624.3 | -307.1 | 11.21 | 0.004** |

| Models – *Acquisition Phase - All conditions* | df | AIC | Log-Lik | χ^2^ | p(>χ^2^) |
| --- | --- | --- | --- | --- | --- |
| Model 1: Score ~ Choice*Condition*Trial*Colour + (1\|BeeID) | 49 | 2650.8 | -1276.4 |  |  |
| Model 2: Score ~ Choice*Condition*Trial + Colour + (1\|BeeID) | 26 | 2640.0 | -1294.0 | 35.13 | 0.051 |
| Model 3: Score ~ Choice*Condition*Trial + (1\|BeeID) | 25 | 2638.0 | -1294.0 | 0 | 1 |
| Model 4: Score ~ Choice*Trial+Condition + (1\|BeeID) | 10 | 2658.1 | -1319.0 | 50.11 | <0.001*** |
| Model 5: Score ~ Choice*Condition+Trial + (1\|BeeID) | 14 | 2653.9 | -1313.0 | 37.94 | <0.001*** |

| Models – *Acquisition Phase – Distances Walked – All Conditions* | df | AIC | Log-Lik | χ^2^ | p(>χ^2^) |
| --- | --- | --- | --- | --- | --- |
| Model 1: Distance ~ Condition*Trial + (1\|BeeID) | 10 | -63.4 | 41.7 |  |  |
| Model 2: Distance ~ Condition + Trial + (1\|BeeID) | 7 | -67.9 | 41.0 | 1.45 | 0.69 |
| Model 3: Distance ~ Trial + (1\|BeeID) | 4 | -68.6 | 38.3 | 5.34 | 0.15 |
| Model 4: Distance ~ 1 + (1\|BeeID) | 3 | -63.7 | 34.9 | 6.86 | 0.009** |

| Models – *Acquisition Phase – Walking Speed – All Conditions* | df | AIC | Log-Lik | χ^2^ | p(>χ^2^) |
| --- | --- | --- | --- | --- | --- |
| Model 1: Speed ~ Condition*Trial + (1\|BeeID) | 10 | -4596.7 | 2308.4 |  |  |
| Model 2: Speed ~ Condition + Trial + (1\|BeeID) | 7 | -4583.5 | 2298.7 | 19.27 | <0.001*** |
| Model 3: Speed ~ Trial + (1\|BeeID) | 4 | -4588.3 | 2298.1 | 1.19 | 0.76 |
| Model 4: Speed ~ 1 + (1\|BeeID) | 3 | -4417.3 | 2211.7 | 172.9 | <0.001*** |

| Models – *Acquisition Phase – Tortuosity – All Conditions* | df | AIC | Log-Lik | χ^2^ | p(>χ^2^) |
| --- | --- | --- | --- | --- | --- |
| Model 1: Tortuosity ~ Condition*Trial + (1\|BeeID) | 10 | 4845.9 | -2413.0 |  |  |
| Model 2: Tortuosity ~ Condition + Trial + (1\|BeeID) | 7 | 4844.5 | -2415.3 | 4.60 | 0.20 |
| Model 3: Tortuosity ~ Trial + (1\|BeeID) | 4 | 4842.1 | -2417.1 | 3.62 | 0.31 |
| Model 4: Tortuosity ~ 1 + (1\|BeeID) | 3 | 4840.3 | -2417.2 | 0.17 | 0.68 |

| Models – *Acquisition Phase – Latency – All Conditions* | df | AIC | Log-Lik | χ^2^ | p(>χ^2^) |
| --- | --- | --- | --- | --- | --- |
| Model 1: Latency ~ Condition*Trial + (1\|BeeID) | 10 | 4973.8 | -2476.9 |  |  |
| Model 2: Latency ~ Condition + Trial + (1\|BeeID) | 7 | 4970.1 | -2478.1 | 2.32 | 0.51 |
| Model 3: Latency ~ Condition + (1\|BeeID) | 6 | 4990.0 | -2489.0 | 21.85 | <0.001*** |
| Model 4: Latency ~ 1 + (1\|BeeID) | 3 | 4985.6 | -2489.8 | 1.67 | 0.65 |

| Models – *Test – All Conditions* | df | AIC | Log-Lik | χ^2^ | p(>χ^2^) |
| --- | --- | --- | --- | --- | --- |
| Model 1: Score ~ Choice*Condition*Colour + (1\|BeeID) | 25 | 298.6 | -124.3 |  |  |
| Model 2: Score ~ Choice*Condition + Colour + (1\|BeeID) | 14 | 293.4 | -132.7 | 16.89 | 0.11 |
| Model 3: Score ~ Choice*Colour + Condition + (1\|BeeID) | 10 | 287.4 | -133.7 | 18.84 | 0.22 |
| Model 4: Score ~ Choice + Condition + Colour + (1\|BeeID) | 8 | 285.8 | -134.9 | 2.39 | 0.30 |
| Model 5: Score ~ Choice + Colour + (1\|BeeID) | 5 | 279.8 | -134.9 | 0 | 1 |
| Model 6: Score ~ Choice + (1\|BeeID) | 4 | 277.8 | -134.9 | 0 | 1 |
| Model 7: Score ~ 1 + (1\|BeeID) | 2 | 290.4 | -143.2 | 16.65 | <0.001*** |

**Table S3**: GLMM Analysis Experiment 3 – Models comparison.

| Models – *Acquisition Phase - Both conditions* | df | AIC | Log-Lik | χ^2^ | p(>χ^2^) |
| --- | --- | --- | --- | --- | --- |
| Model 1: Score ~ Choice*Colour*Trial*Condition + (1\|BeeID) | 25 | 2180.4 | -1065.2 |  |  |
| Model 2: Score ~ Choice*Colour*Trial + Condition + (1\|BeeID) | 14 | 2177.3 | -1074.7 | 18.93 | 0.06 |
| Model 3: Score ~ Choice*Colour*Trial + (1\|BeeID) | 13 | 2175.3 | -1074.7 | 0 | 1 |
| Model 4: Score ~ Choice*Colour + Trial + (1\|BeeID) | 8 | 2229.7 | -1106.8 | 64.33 | <0.001*** |
| Model 5: Score ~ Choice*Trial + Colour+ (1\|BeeID) | 8 | 2179.9 | -1082.0 | 14.58 | 0.012* |

| Models – *Acquisition Phase – Blue +* | df | AIC | Log-Lik | χ^2^ | p(>χ^2^) |
| --- | --- | --- | --- | --- | --- |
| Model 1: Score ~ Choice* Trial + (1\|BeeID) | 7 | 1190.4 | -588.2 |  |  |
| Model 2: Score ~ Choice + Trial + (1\|BeeID) | 5 | 1235.2 | -612.6 | 48.86 | <0.001*** |

| Models – *Acquisition Phase – Green +* | df | AIC | Log-Lik | χ^2^ | p(>χ^2^) |
| --- | --- | --- | --- | --- | --- |
| Model 1: Score ~ Choice* Trial + (1\|BeeID) | 7 | 987.0 | -486.5 |  |  |
| Model 2: Score ~ Choice + Trial + (1\|BeeID) | 5 | 998.42 | -494.2 | 15.47 | <0.001*** |

| Models – *Acquisition Phase – Walking Speed – Both Conditions* | df | AIC | Log-Lik | χ^2^ | p(>χ^2^) |
| --- | --- | --- | --- | --- | --- |
| Model 1: Speed ~ Condition*Trial + (1\|BeeID) | 6 | -4090.4 | 2051.2 |  |  |
| Model 2: Speed ~ Condition + Trial + (1\|BeeID) | 5 | -4092.0 | 2051.0 | 0.39 | 0.53 |
| Model 3: Speed ~ Condition + (1\|BeeID) | 4 | -4008.8 | 2008.4 | 85.20 | <0.001*** |
| Model 4: Speed ~ Trial + (1\|BeeID) | 4 | -4088.0 | 2048.0 | 6.03 | 0.014* |

| Models – *Acquisition Phase – Distances Walked – Both Conditions* | df | AIC | Log-Lik | χ^2^ | p(>χ^2^) |
| --- | --- | --- | --- | --- | --- |
| Model 1: Distance ~ Condition*Trial + (1\|BeeID) | 6 | -85.8 | 48.9 |  |  |
| Model 2: Distance ~ Condition + Trial + (1\|BeeID) | 5 | -86.6 | 48.3 | 1.22 | 0.27 |
| Model 3: Distance ~ Condition + (1\|BeeID) | 4 | -87.2 | 47.6 | 1.45 | 0.23 |
| Model 4: Distance ~ 1 + (1\|BeeID) | 3 | -81.7 | 43.9 | 7.45 | 0.006** |

| Models – *Acquisition Phase – Tortuosity – Both Conditions* | df | AIC | Log-Lik | χ^2^ | p(>χ^2^) |
| --- | --- | --- | --- | --- | --- |
| Model 1: Tortuosity ~ Condition*Trial + (1\|BeeID) | 6 | 3639.9 | -1813.9 |  |  |
| Model 2: Tortuosity ~ Condition + Trial + (1\|BeeID) | 5 | 3638.0 | -1814.0 | 0.07 | 0.79 |
| Model 3: Tortuosity ~ Trial + (1\|BeeID) | 4 | 3636.5 | -1814.3 | 0.56 | 0.45 |
| Model 4: Tortuosity ~ 1 + (1\|BeeID) | 3 | 3642.5 | -1818.2 | 7.95 | 0.0048** |

| Models – *Acquisition Phase – Latency – Both Conditions* | df | AIC | Log-Lik | χ^2^ | p(>χ^2^) |
| --- | --- | --- | --- | --- | --- |
| Model 1: Latency ~ Condition*Trial + (1\|BeeID) | 6 | 6668.5 | -3328.3 |  |  |
| Model 2: Latency ~ Condition + Trial + (1\|BeeID) | 5 | 6666.6 | -3328.3 | 0.06 | 0.80 |
| Model 3: Latency ~ Condition + (1\|BeeID) | 4 | 6666.6 | -3329.3 | 1.97 | 0.16 |
| Model 4: Latency ~ Trial + (1\|BeeID) | 3 | 6664.8 | -3328.4 | 0.19 | 0.66 |
| Model 5: Latency ~ 1 + (1\|BeeID) | 3 | 6664.7 | -3329.4 | 1.97 | 0.16 |

| Models – *Test – Both Conditions* | df | AIC | Log-Lik | χ^2^ | p(>χ^2^) |
| --- | --- | --- | --- | --- | --- |
| Model 1: Score ~ Choice*Condition*Colour + (1\|BeeID) | 13 | 240.5 | -107.2 |  |  |
| Model 2: Score ~ Choice*Condition + Colour + (1\|BeeID) | 8 | 238.4 | -111.2 | 7.86 | 0.16 |
| Model 3: Score ~ Choice*Colour + Condition + (1\|BeeID) | 8 | 237.2 | -110.6 | 6.72 | 0.24 |
| Model 4: Score ~ Choice + Condition + Colour + (1\|BeeID) | 6 | 235.2 | -111.6 | 1.99 | 0.37 |
| Model 5: Score ~ Choice + Colour + (1\|BeeID) | 5 | 233.2 | -111.6 | 0 | 1 |
| Model 6: Score ~ Choice + (1\|BeeID) | 4 | 231.2 | -111.6 | 0 | 1 |
| Model 7: Score ~ 1 + (1\|BeeID) | 2 | 259.9 | -127.9 | 32.7 | <0.001*** |
